# Brainstem neurochemical profiles after hospitalisation for COVID-19: a 7T MR spectroscopy study

**DOI:** 10.3389/fnins.2025.1617709

**Published:** 2025-10-15

**Authors:** Carina Graf, Betty Raman, Anne Manktelow, Doris A. Chatfield, William T. Clarke, Catarina Rua, Virginia F. J. Newcombe, Victoria C. Lupson, Stephen J. Sawcer, Joanne G. Outtrim, Karen D. Ersche, Lin Qiu, Martyn Ezra, Rory McDonald, Stuart Clare, Mark Philip Cassar, Stefan Neubauer, Edward T. Bullmore, David K. Menon, James B. Rowe, Kyle Pattinson, Christopher T. Rodgers, the Cambridge NeuroCOVID group, Kieren Allinson, Fahim Anwar, Junaid Bhatti, Edward T Bullmore, Doris A Chatfield, David Christmas, Alasdair J Coles, Jonathan P Coles, Marta Correia, Tilak Das, Anne Elmer, Paul C Fletcher, Alasdair W Jubb, Victoria C Lupson, Anne Manktelow, David K Menon, Andrew Michell, Edward Needham, Virginia FJ Newcombe, Joanne G Outtrim, Linda Pointon, Christopher T Rodgers, James B Rowe, Catarina Rua, Stephen J Sawcer, Nyarie Sithole, Lennart RB Spindler, Emmanuel A Stamatakis, Jonathon Taylor, Fernanda Valerio, Barry Widmer, Guy B Williams, the CITIID-NIHR COVID-19 BioResource Collaboration, John Allison, Gisele Alvio, Stephen Baker, Sharon Baker, Laura Bergamashi, Nonantzin Beristain-Covarrubias, Areti Bermperi, Ariana Betancourt, Heather Biggs, Lucy Booth, Rebecca Boston, Georgie Bower, John Bradley, Karen Brookes, Ashlea Bucke, Ben Bullman, Helen Butcher, Sarah L. Caddy, Jo Calder, Laura G. Caller, Laura Canna, Daniela Caputo, Matt Chandler, Yasmin Chaudhry, Patrick Chinnery, Debbie Clapham-Riley, Daniel Cooper, Chiara Cossetti, Cherry Crucusio, Isabel Cruz, Martin D. Curran, Ranalie de Jesus, Aloka De Sa, Katie Dempsey, Eleanor Dewhurst, Giovanni Di Stephano, Jason Domingo, Gordon Dougan, Benjamin J. Dunmore, Anne Elmer, Maddie Epping, Stuart Fawke, Theresa Feltwell, Christian Fernandez, Alexander Ferreira, Stewart Fuller, Anita Furlong, Iliana Georgana, Ian Goodfellow, Stefan Gräf, Barbara Graves, Jennifer Gray, Richard Grenfell, Ravindra Gupta, Thevinya Gurugama, Lihinya Gurugama, Grant Hall, William L. Hamilton, Julie Harris, Sabine Hein, Sarah Hewitt, Andrew Hinch, Josh Hodgson, Emily C. Horner, Myra Hosmillo, Zhaleh Hosseini, Charlotte J. Houldcroft, Christopher Huang, Robert Hughes, Oisin Huhn, Kelvin Hunter, Tasmin Ivers, Rhys Izuagbe, Aminu S. Jahun, Isobel Jarvis, Heather Jones, Emma Jones, Sherly Jose, Maša Josipović, Mary Kasanicki, Jane Kennet, Fahad A. Khokhar, Yvonne King, Rebecca King, Nathalie Kingston, Jenny Kourampa, Anna G. Kovalenko, Emma Le Gresley, Ekaterina Legchenko, Paul J. Lehner, Daniel Lewis, Emily Li, Rachel Linger, Paul A. Lyons, Joe Marsden, Jennifer Martin, Nicholas J. Matheson, Caroline McMahon, Anne Meadows, Sarah Meloy, Vivien Mendoza, Luke W. Meredith, Federica Mescia, Alexei Moulton, Francesca Muldoon, Thomas Mulroney, Criona O’Brien, Ciara O’Donnell, Charmain Ocaya, Ommar Omarjee, Nigel Ovington, Sofia Papadia, Roxana Paraschiv, Surendra Parmar, Ciro Pasquale, Christopher J. Penkett, Marlyn Perales, Marianne Perera, Isabel Phelan, Malte L. Pinckert, Linda Pointon, Petra Polgarova, Nicole Pond, Jane Price, Venkatesh Ranganath, Rebecca Rastall, Carla Ribeiro, Nathan Richoz, Nika Romashova, Jane Rowlands, Valentina Ruffolo, Maria Rust, Abigail Sage, Jennifer Sambrook, Caroline Saunders, Natalia Savoinykh, Ingrid Scholtes, Katherine Schon, Mayurun Selvan, Rahul Sharma, Joy Shih, Kenneth G. Smith, Sarah Spencer, Hannah Stark, Kathleen E. Stirrups, Mateusz Strezlecki, Charlotte Summers, Rachel Sutcliffe, James Thaventhiran, Tobias Tilly, Zhen Tong, Hugo Tordesillas, M. Estee Torok, Mark R. Toshner, Paul Townsend, Carmen Treacy, Lori Turner, Phoebe Vargas, Bensi Vergese, Neil Walker, Laura Watson, Jennifer Webster, Michael P. Weekes, Kate Williamson, Jennifer Wood, Jieniean Worsley, Marta Wylot, Anna Yakovleva, Juan Carlos Yam-Puc, Julie-Ann Zerrudo

**Affiliations:** ^1^Wolfson Brain Imaging Centre, Department of Clinical Neurosciences, University of Cambridge, Cambridge, United Kingdom; ^2^Division of Cardiovascular Medicine, Radcliffe Department of Medicine, Oxford University Hospitals NHS Foundation Trust, University of Oxford, Oxford, United Kingdom; ^3^Division of Anaesthesia, Department of Medicine, University of Cambridge, Cambridge, United Kingdom; ^4^Wellcome Centre for Integrative Neuroimaging, FMRIB, Nuffield Department of Clinical Neurosciences, John Radcliffe Hospital, University of Oxford, Oxford, United Kingdom; ^5^Perceptive, London, United Kingdom; ^6^Cambridge Centre for Parkinson-Plus, University of Cambridge, Cambridge, United Kingdom; ^7^Department of Clinical Neurosciences, University of Cambridge, Cambridge, United Kingdom; ^8^Department of Psychiatry, University of Cambridge, Cambridge, United Kingdom; ^9^Department of Addictive Behaviour and Addiction Medicine, Central Institute of Mental Health, University of Heidelberg, Mannheim, Germany; ^10^Nuffield Department of Clinical Neurosciences, University of Oxford, Oxford, United Kingdom; ^11^Cambridge University Hospitals NHS Foundation Trust, University of Cambridge, Cambridge, United Kingdom; ^12^MRC Cognition and Brain Sciences Unit, University of Cambridge, Cambridge, United Kingdom

**Keywords:** COVID-19, brainstem, 7T, magnetic resonance spectroscopy, neuroinflammation

## Abstract

**Background:**

Somatic, cognitive and mental health issues have been identified in three-quarters of people 5 months after hospitalisation for severe acute SARS-CoV-2 (COVID-19) infection. The underlying neuroanatomical basis of these symptoms remains unclear, but recent studies suggest a role for altered brainstem physiology. We aimed to test the hypothesis that brainstem neurochemical profiles differ in patients who had been hospitalised for COVID-19 compared to matched controls using 7T magnetic resonance spectroscopy (MRS).

**Methods:**

This prospective case–control study recruited 34 individuals who were hospitalised for COVID-19 and 15 healthy controls with no history of COVID-19 infection from two major UK hospitals before vaccines became available. The participants underwent 7T semi-adiabatic localization by adiabatic selective refocusing (sLASER) ^1^H-MRS at the ponto-medullary junction. Water-referenced metabolite concentrations were compared between the patients and controls and correlated with infection severity, as measured by maximum C-reactive protein (CRP_max_) assay during inpatient admission. Linear mixed modelling was used with a 0.05 significance level.

**Results:**

Spectral quality was high/acceptable in 44/49 participants according to the MRS Consensus criteria. The magnitude of inflammation during patient admission (i.e., CRP_max_) correlated positively with *myo*-inositol concentration (*β* = 0.005, *p* = 0.035), as did patient-reported symptoms (*β* = −0.564, *p* = 0.023). However, metabolite concentrations were not significantly different between the patients and controls.

**Conclusion:**

We show the feasibility of assessing brainstem neurochemical profiles using 7T ^1^H-MRS in a multi-centre study. Technical limitations at one site’s 7T MRI led to variable repetition times, which limited our statistical power and should be avoided in future studies. Our findings highlight the need for further investigation into the role of neuroinflammation in post-acute COVID-19.

## Introduction

Patients hospitalised with severe acute SARS-CoV-2 coronavirus infection (COVID-19) sometimes report persistent symptoms weeks to months after discharge. These symptoms include fatigue and breathlessness, cognitive deficits and mental health problems—such as anxiety, depression and post-traumatic stress disorder (Nalbandian et al., 2021).

The neuroanatomical effects of severe acute SARS-CoV-2 infection over this timescale are uncertain, but accumulating evidence suggests a role for altered brainstem microarchitecture and neurochemistry in key brainstem structures (Cavallieri et al., 2022). SARS-CoV-2 is thought to promote neuroinflammation, and it may even be neuroinvasive in the brainstem (Mao et al., 2020; Li et al., 2020; Meinhardt et al., 2021; Song et al., 2021; Matschke et al., 2020). Diffusion tensor imaging shows changes in the brainstem in the acute phase (Newcombe et al., 2021). Circulating biomarkers of brain injury remain elevated months after acute illness, and brainstem susceptibility is increased (Needham et al., 2022; Rua et al., 2024).

We used ultra-high field magnetic resonance spectroscopy (7T MRS) with the semi-adiabatic localization by adiabatic selective refocusing (sLASER) sequence as a quantitative non-invasive method to assess brain metabolite concentrations (Kreis et al., 2020). Based on evidence of possible brainstem inflammation as a mechanistic process, our primary analysis explored differences in *myo*-inositol concentrations between patients and controls, as *myo*-inositol is recognised as a biomarker for neuroinflammation (Chang et al., 2013). In addition, we undertook exploratory analyses of other metabolites. Specifically, we also measured concentrations of the following: N-acetyl aspartate, a marker of mitochondrial function and neuronal integrity; glutamate and glutamine, which play a crucial role in maintaining neurotransmitter homeostasis and synaptic transmission (Tani et al., 2014); and *γ*-Aminobutyric acid, a key inhibitory neurotransmitter. In addition, both Gln and GABA also have metabolic functions and pools that are not fully separable from their neurotransmitter function by ^1^H-MRS (Duarte, 2025). We also measured choline-containing compounds, a marker of membrane turnover (Oz et al., 2014; Eichler et al., 2009; Kruse et al., 1993). These exploratory analyses were not corrected for multiple comparisons.

Previous studies have shown that 7T MRS quantifies neurochemical profiles more precisely than 3 T in the cerebrum and cerebellum (van de Bank et al., 2015; Terpstra et al., 2016). One 7T study using a custom coil with 16-channel parallel transmit showed high-quality brainstem spectra (Joers et al., 2018). We believe ours is the first 7T MRS study of the brainstem using commercially available 7T hardware.

Our aim was to test whether patients hospitalised with COVID-19 show persistent derangement in metabolism—especially involving *myo*-inositol—around the ponto-medullary junction area of the brainstem, which is the site of key neuromodulatory nuclei and respiratory control centres.

## Methods

### Participants

**Figure 1:**
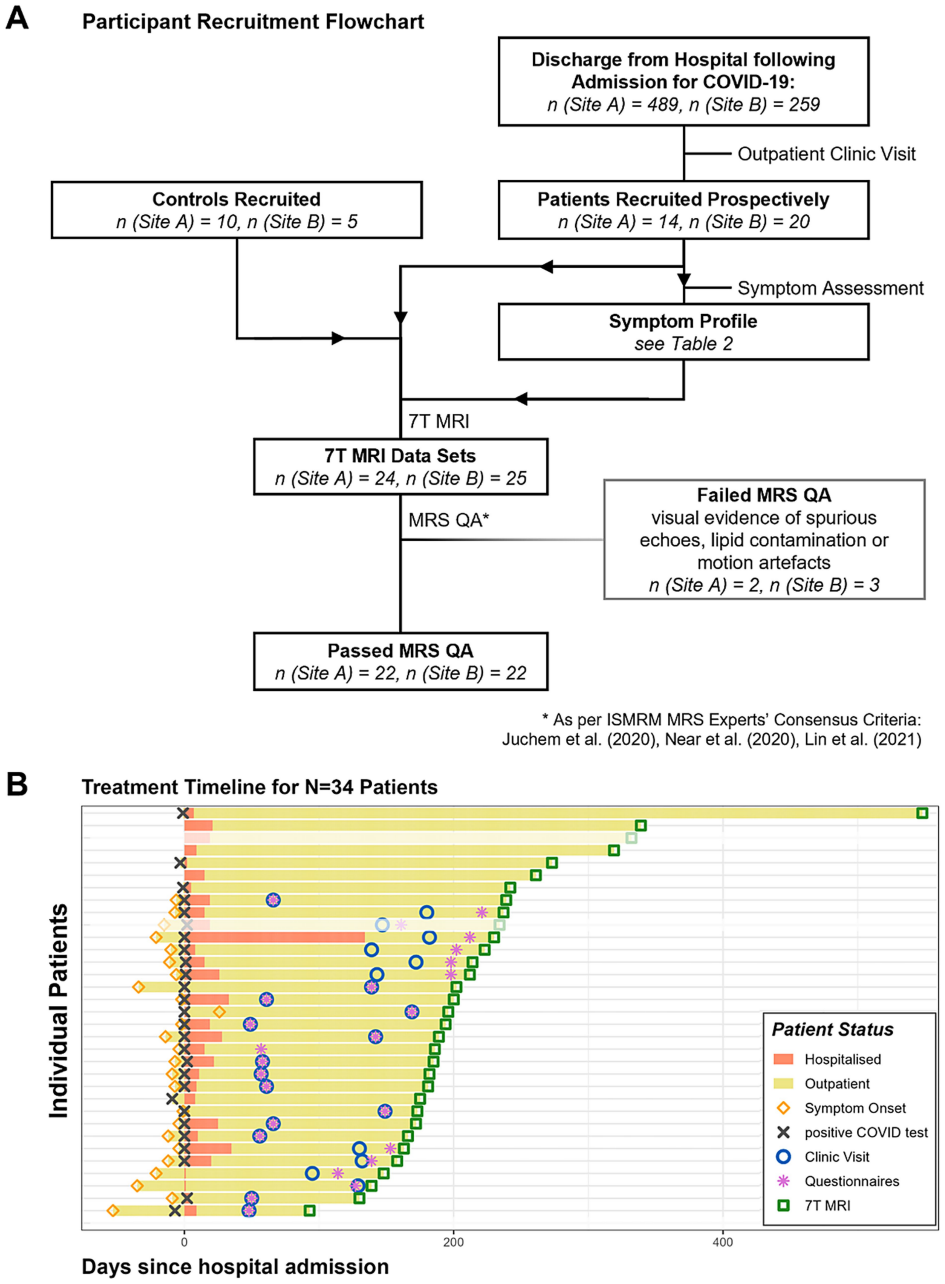
**(A)** Recruitment flow chart and **(B)** patient timeline. **(A)** Flow diagram of patient and control recruitment and magnetic resonance spectroscopy (MRS) exclusion criteria at both sites. A total of 34 COVID-19 patients and an additional 15 healthy controls were recruited. **(B)** Timeline of data collection for patients relative to the day of hospital admission. Participants displayed in faint colours later failed to meet minimal MRS quality standards and were excluded from further analysis. MRS, magnetic resonance spectroscopy; QA, quality assurance.

Patients were prospectively recruited following hospitalisation for acute COVID-19 infection at Cambridge University Hospitals NHS Foundation Trust (Site A) and Oxford University Hospitals NHS Foundation Trust (Site B). Patients were admitted to the hospital between 4 March 2020 and 21 February 2021, that is, during the first and second waves of COVID-19 infection in the UK, before vaccines became broadly available. We included patients aged ≥18 who were acutely hospitalised for COVID-19, confirmed by a positive reverse transcriptase–polymerase chain reaction test and clinical history (Figure 1A). Participants were excluded if they had a safety contraindication to 7T MRI, any signs of active COVID-19 infection on the day of MRI imaging, any significant pre-existing cardiac, brain or liver disease or end-stage renal failure. Activity at Site A was approved by the Cambridgeshire Research Ethics Committee (REC 97/290), the NIHR BioResource (REC 17/EE/0025) and the Norfolk REC (12/EE/0395). Activity at Site B was approved by the North-West Preston Research Ethics Committee (20/NW/0235). The participants provided informed written consent to participate in this study. Healthy controls were recruited between October 2020 and April 2021 to be age- and sex-matched, mainly via the NIHR BioResource collaboration and also from volunteers within the imaging community. All controls were screened for symptoms of respiratory viral illness or a history of contact with infected individuals during the pandemic. Screening included symptom questionnaires completed 48 h prior to the scan and repeated on the day of scanning. Only individuals with no history of respiratory illness symptoms during the pandemic and no recent exposure to COVID-19 were enrolled for 7T MRI control scans. Site A recruited 14 patients (nine female, five male), with a median age of 52 years (range 32–70 years), and 10 healthy controls who had never tested positive for COVID-19 (six female, four male) via the NIHR BioResource collaboration. Site B recruited 20 patients (five female, 15 male), with a median age of 57 years (range 22–78 years), and five healthy controls who had never tested positive for COVID-19 (two female, three male) (Table 1).

**Table 1:** Participant demographics.

Participants	Site A					Site B					Both sites				
	Control		Patients		*p*	Control		Patients		*p*	Controls		Patients		*p*
	*n* (%)	Median (IQR)	*n* (%)	Median (IQR)		*n* (%)	Median (IQR)	*n* (%)	Median (IQR)		*n* (%)	Median (IQR)	*n* (%)	Median (IQR)	
*N* (enrolled) (female)	10 (6F)		14 (9F)		1*	5 (2F)		20 (5F)		0.6*	15 (8F)		34 (14F)		0.5*
N (analysed) (female)	9 (5F)		13 (8F)		1*	4 (1F)		18 (4F)		1*	13 (6F)		31 (12F)		0.7*
Demographics															
Age (years)	9 (100)	48.0 (17.0)	13 (100)	51.0 (9.0)	0.3^†^	4 (100)	62.5 (8.5)	18 (100)	57.0 (15.5)	0.9^†^	13 (100)	50.0 (25.0)	31 (100)	56.0 (12.0)	0.2^†^
Body mass index (kg/m^2^)	9 (100)	24.4 (3.6)	13 (100)	29.1 (4.1)	0.017^†^	4 (100)	23.4 (4.1)	16 (89)	27.1 (10.3)	0.053^†^	13 (100)	24.4 (4.2)	29 (94)	28.9 (8.0)	0.001^†^

*The *p*-value from Fisher’s exact test (Fisher, 1922), ^†^the *p*-value from a two-sided *t*-test. Values are reported as medians and interquartile ranges (IQRs) where applicable.

#### Assessment of COVID-19 severity

We assessed COVID-19 severity using the World Health Organization (WHO) Ordinal Scale for Clinical Improvement (UN World Health Organization, 2020), which rates disease severity from 0 (uninfected) to 8 (dead). For ease of reference, this scale is reproduced in Supplementary Figure 1. Across both sites, 16 patients had experienced mild to moderate disease (maximum severity score ≤ 4), whilst 15 had been severely ill (maximum severity score >4) and had been admitted to the intensive care unit for at least 1 day. We also assessed the magnitude of the inflammatory host response during the acute phase of the disease as the highest assay result for C-reactive protein (CRP) recorded during the period of hospital admission (Table 2).

**Table 2:** Disease severity and symptom profile information at follow-up for the patients included in the analysis.

COVID clinical presentation	Site A		Site B		Both sites	
	*n* (%)	Median (IQR)	*n* (%)	Median (IQR)	*n* (%)	Median (IQR)
WHO Disease Severity (0–8):	13 (100)	4.0 (5.0)	18 (100)	4.5 (1.8)	31 (100)	4.0 (2.5)
N_mild_, _moderate_ (severity ≤4)	7 (54)	–	9 (50)	–	16 (52)	–
N_Severe_ (severity >4)	6 (46)	–	9 (50)	–	15 (48)	–
Highest CRP during admission: CRP_max_ (mg/dL)	13 (100)	87.0 (282.0)	14 (78)	192.6 (92.8)	27 (87)	182.7 (231.4)
Days in hospital (days)	13 (100)	15 (25)	18 (100)	10.5 (10.8)	31 (100)	11 (14.5)
Time from hospital admission to 7T MRI scan (days)	13 (100)	196 (51)	18 (100)	190.0 (79.8)	31 (100)	194.0 (61.0)
Time from clinic visit to 7T MRI scan (days)	13 (100)	47 (30)	10 (56)	122.5 (29)	23 (74)	63 (71.5)
Patient symptom profile at follow-up						
PHQ-9 (0–27)	13 (100)	7.0 (10.0)	18 (100)	4.5 (5.5)	31 (100)	6.0 (7.0)
GAD-7 (0–21)	13 (100)	4.0 (5.0)	18 (100)	2.0 (5.0)	31 (100)	3.0 (4.0)
SF-36 (100–0)						
Physical functioning	12 (92)	47.5 (47.5)	16 (89)	67.5 (47.5)	28 (90)	62.5 (55.0)
Role limitations – Physical	11 (85)	0.0 (62.5)	16 (89)	12.5 (100.0)	27 (87)	0.0 (100.0)
Bodily pain	11 (85)	45.0 (61.5)	16 (89)	67.0 (25.5)	27 (87)	62.0 (44.0)
General health	11 (85)	55.0 (22.5)	16 (89)	61.0 (31.8)	27 (87)	60.0 (28.5)
Energy/Vitality	11 (85)	55.0 (27.5)	16 (89)	47.5 (28.8)	27 (87)	50.0 (32.5)
Social functioning	11 (85)	62.0 (24.5)	16 (89)	56.2 (40.6)	27 (87)	62.0 (31.2)
Role limitations – Emotional	11 (85)	33.0 (100.0)	16 (89)	33.3 (75.0)	27 (87)	33.3 (100.0)
Mental health	11 (85)	76.0 (20.0)	16 (89)	76.0 (21.0)	27 (87)	76.0 (20.0)
Post 6MWT breathlessness (0–10)	13 (100)	2.0 (3.0)	17 (94)	3.0 (3.0)	30 (97)	2.8 (3.5)

Disease severity was assessed using the COVID-19 World Health Organization (WHO) ordinal scale (range 0–8) and the highest C-reactive protein (CRP) assay result obtained during the period of hospital admission, CRP_max_. The patients’ general physical, cognitive and mental dysfunction at follow-up were assessed using the 9-item Patient Health Questionnaire (PHQ-9), Generalized Anxiety Disorder 7-item scale (GAD-7) and 36-item Short Form Health Survey (SF-36), as well as a 6-min walking test (6MWT). 6MWT, 6 min walking test; CRP, C-reactive protein; GAD-7, Generalized Anxiety Disorder 7-item scale; IQR, interquartile range; PHQ-9, Patient Health Questionnaire-9; SF-36, Short Form-36; WHO, World Health Organization.

#### Assessment of post-acute symptoms

The patients were assessed for cognitive and clinical symptoms of post-acute COVID-19 during their outpatient clinical follow-up (Figure 1B). This included a 6-min walking test and questionnaires designed to quantify aspects of physical and mental health. Specifically, these included the Generalized Anxiety Disorder 7-item scale (Swinson, 2006; Spitzer et al., 2006), the 9-item Patient Health Questionnaire (Kroenke et al., 2001) and the 36-item Short Form Health Survey (SF-36) (Ware Jr and Sherbourne, 1992). To minimise patient fatigue from multiple tests and surveys, we limited the questionnaire assessment to a single time point within 1 year of the 7T scan. For the analysis, we extracted relevant sections from the SF-36 responses to generate subscores quantifying physical functioning, role limitations due to physical health, role limitations due to emotional problems, energy/vitality levels, mental health, social functioning, bodily pain and general health. To harmonise interpretation, we used *inverted* anxiety and health scores in our analysis so that higher values consistently represent better mental health.

### Magnetic resonance methods

The patients underwent 7T MRI and MRS between 85 and 542 days post-discharge from the hospital (median(IQR) = 173 (66) days). Site A used a 7T Terra MRI system (Siemens, Germany), and Site B used a Magnetom 7T MRI system (Siemens, Germany). The sites used identical 1Tx/32Rx head coils (Nova Medical Inc., USA). The protocol comprised the following: localiser imaging, a 0.7mm^3^ isotropic structural scan (MP2RAGE, 2.64 ms TE, 725/2150 ms TIs, 3,500 ms TR, Supplementary Table 1) (Clarke et al., 2020); resting-state fMRI; quantitative susceptibility imaging and sLASER MRS (Oz and Tkáč, 2011; Deelchand et al., 2021), which is the focus of this article. The imaging results of this patient cohort are being reported separately (Rua et al., 2024).

#### Spectroscopy

Brainstem metabolism was assessed using sLASER 7T-MRS (MRS package v2017-07, University of Minnesota, USA). A 12 x 12 x 20 mm^3^ (2.9 mL) voxel was centred on the ponto-medullary junction and rotated in the foot–head direction so that it was parallel to the medulla. This location covers key neuromodulatory nuclei and respiratory control centres. The sLASER acquisition consisted of 120 water-suppressed signal averages, a 26 ms TE and GOIA-WURST refocusing (Oz and Tkáč, 2011; Deelchand et al., 2021). FASTMAP B_0_ shimming was employed. Excitation and water suppression flip angles were adjusted using a parameter sweep pre-scan (Murley et al., 2020). Two spectra (NA = 2 each) were acquired without water suppression as a water concentration reference and to correct for eddy currents. The 7T Terra MRI at Site A followed the International Electrotechnical Committee (IEC) 3rd revision specific absorption rate (SAR) limits, which permitted a fixed 5-s repetition time (TR) for all participants. The Magnetom 7T MRI at Site B followed the IEC 2nd revision SAR limits, meaning that repetition times varied from 5.0 to 7.7 s at Site B. Further details are provided in Supplementary Table 2.

#### Post-processing

##### Imaging

MP2RAGE structural images underwent phase-sensitive reconstruction (Mougin et al., 2016), N4 bias-field correction, brain extraction and tissue segmentation (grey matter, white matter and CSF) using ANTs (v2.1.0) and SPM12 (Avants et al., 2014; Penny et al., 2007; Rua et al., 2020).

##### Structural image registration

The structural images were registered to MNI standard space in three steps: (1) rigid, (2) affine and (3) symmetric image normalisation (Avants et al., 2008). We used a mutual information metric for the first two steps, and cross-correlation for the third step. Each step was run with four spatial downsampling levels.

#### Data analysis

##### Voxel placement consistency

Voxel placement consistency was assessed by calculating the Sørensen–Dice coefficient for each volume of interest relative to the mean volume of interest across all participants in standard space. The Sørensen–Dice coefficient (DSC) for each participant’s voxel was defined in MNI standard space as:


(1) $$\mathrm{DSC}=\frac{2\times \text{Area of overlap}}{\text{Total Area}}$$

##### CSF fraction

Volumetric CSF fraction (f_CSF_) was calculated from the same coregistered structural images.

##### Spectroscopy

Spectroscopy data were converted to NIfTI-MRS format using spec2nii v0.7.0 (Clarke et al., 2022) and then analysed with FSL-MRS v2.1.12 (Clarke et al., 2021). FSL-MRS is a modern, open-source spectroscopy analysis tool included in the widely used FMRIB Software Library (Jenkinson et al., 2012). It implements linear combination modelling to fit MR spectra. It uses the Brain Imaging Data Structure (Gorgolewski et al., 2016) and NIfTI formats (Clarke et al., 2022). FSL-MRS has been validated against other spectral fitting tools (Clarke et al., 2020). Pre-processing used fsl_mrs_prepoc to implement the following pipeline: frequency alignment of transients; removal of anomalous transients (differing by more than 2.58x standard deviations from the median signal within the range of 0.2–4.2 ppm); phase correction of transients; averaging transients to produce a combined free induction decay for each measurement; removal of residual water signal using Hankel Lanczos’ singular value decomposition method and eddy-current correction. We manually checked that transients showing spurious echoes, lipid contamination or motion artefacts were excluded before averaging. Quantitative thresholds for inclusion were as follows: (1) a minimum metabolite signal-to-noise ratio of N-acetyl aspartate ≥ 35; (2) a linewidth, measured as full-width at half maximum, of less than 20 Hz and (3) a linewidth of less than 13 Hz for the unsuppressed water signal. These criteria included transients that were “acceptable” or better according to the published MRS Expert Consensus guidelines for the pre-frontal cortex (Juchem et al., 2021).

Spectra were fitted using the Metropolis–Hastings (MH) algorithm, as described in the Supporting Information. We used a measured macromolecule baseline and simulated basis spectra (Terpstra et al., 2016). Subsequent statistical analysis was based on the fitted folded-normal mean amplitudes and standard deviations.

##### Quantitation

The water referenced metabolite concentrations were corrected for the volumetric fraction of CSF, f_CSF_, within the voxel and globally scaled by the estimated visible water content (Christiansen et al., 1994). The resulting metabolite concentrations are reported in molar units (mmol/L).

In addition, fitted metabolite signal amplitudes were also scaled relative to the fitted total creatine (tCr) signal amplitude to yield unitless concentration ratios. These ratios were included by convention and for ease of comparison with clinical MRS studies.

##### Effect of T_1_ correction

Site B’s 7T MRI system followed the IEC 2nd revision SAR limits, which do not support First Level mode, whereas Site A’s 7T MRI system followed the IEC 3rd revision SAR limits, which allow 2x power in First Level mode. As a result, sometimes Site B could not achieve the study’s planned TR. The resulting variable, and often longer, repetition times at Site B could have caused varying partial saturation effects between scans, disproportionately affecting metabolites with longer T_1_s. There are no published reference values for metabolite T_1_ values in the brainstem at 7T. In an effort to understand the impact of this variation in repetition time, we tried correcting based on published *cortical* T_1_ values.

### Statistical analysis

#### Analysis of between-site differences

Two-sided *t*-tests were used to assess differences between the sites in the combined patient and control groups (Student, 1908; Fisher, 1922) across measures of spectral quality (signal-to-noise ratio, linewidth), voxel placement consistency (Sørensen–Dice coefficients, f_CSF_) and metabolite concentrations. Differences between the groups (i.e., controls vs. patients or Site A vs. Site B) were calculated as follows:


(2) $$\text{difference}=200\%\times \frac{\mu_{\text{Group}\;2}-\mu_{\text{Group}\;1}}{\mu_{\text{Group}\;2}+\mu_{\text{Group}\;1}}$$

Where μ_Group i_ is the group mean for a given quality metric or metabolite concentration.

#### Impact of COVID-19 on metabolite concentrations

We investigated the impact of COVID-19 on the patients’ metabolite concentrations using two separate linear mixed models in R. Each model included either clinical markers or cohort group (patient or control) as fixed effects, with site, age and sex included as random effects.

##### Model 1: effect of disease severity on metabolite concentrations

We included “site” as a random factor in a hierarchical linear mixed model using the lmerTest package (v3.1–3) (Kuznetsova et al., 2017) in R (R Core Team, 2023) to account for the variable TR at Site B resulting from the IEC 2nd revision SAR limits. To investigate the impact of peak disease severity, as measured by the inflammatory marker CRP, we fitted a linear mixed model with markers for peak disease severity as a fixed effect for the patients only. The model was specified as follows:


(3) [met]~Clinical Marker+(1∣site)+(1∣age)+(1∣sex)

Where Clinical Marker represents the highest CRP assay result during hospitalisation.

##### Model 2: investigation of differences between the patients and controls

To identify concentration differences between the patients and controls, we modelled their respective groups as a fixed effect variable (patient), with age, sex and site included as random effects. The model was specified as follows:


(4) [met]~patient+(1∣site)+(1∣age)+(1∣sex)

In all models, missing data points (see Table 1), were assumed to be missing at random and included in statistical modelling employing the Restricted Maximum Likelihood (REML) method. Raw, uncorrected *p*-values were reported.

#### Impact of physical and mental wellbeing at follow-up

As an additional hypothesis-generating analysis, we investigated the impact of physical and mental health on metabolite concentrations at follow-up. We first performed principal component analysis on the questionnaire and breathlessness data for dimensionality reduction (using prcomp in R). The analysis included centred (mean = 0) and scaled (sd = 1) components of the inverted anxiety, health, breathlessness scores and the SF-36 subscores from the patients who had completed all assessments (n = 29). Overall physical and mental health were represented by the first and second principal components. Their impact on metabolite concentrations was investigated by fitting two separate linear mixed models, as described in Equation (3), with each principal component included as the clinical marker:


(5) [met]~Behavioural/FunctionalPC+(1∣site)+(1∣age)+(1∣sex)

## Results

Participant demographic details are shown in Table 1. The patients were hospitalised for a median of 11 days (IQR = 14.5 days, Figure 1B), with a median WHO severity score of 4 across both sites (IQR = 2.5). The 7T MRI data were acquired 194 days (median, IQR = 61 days) after initial hospitalisation and 63 days (median, IQR = 71.5 days) following their outpatient follow-up clinic visit (Table 2).

### Data quality

**Figure 2:**
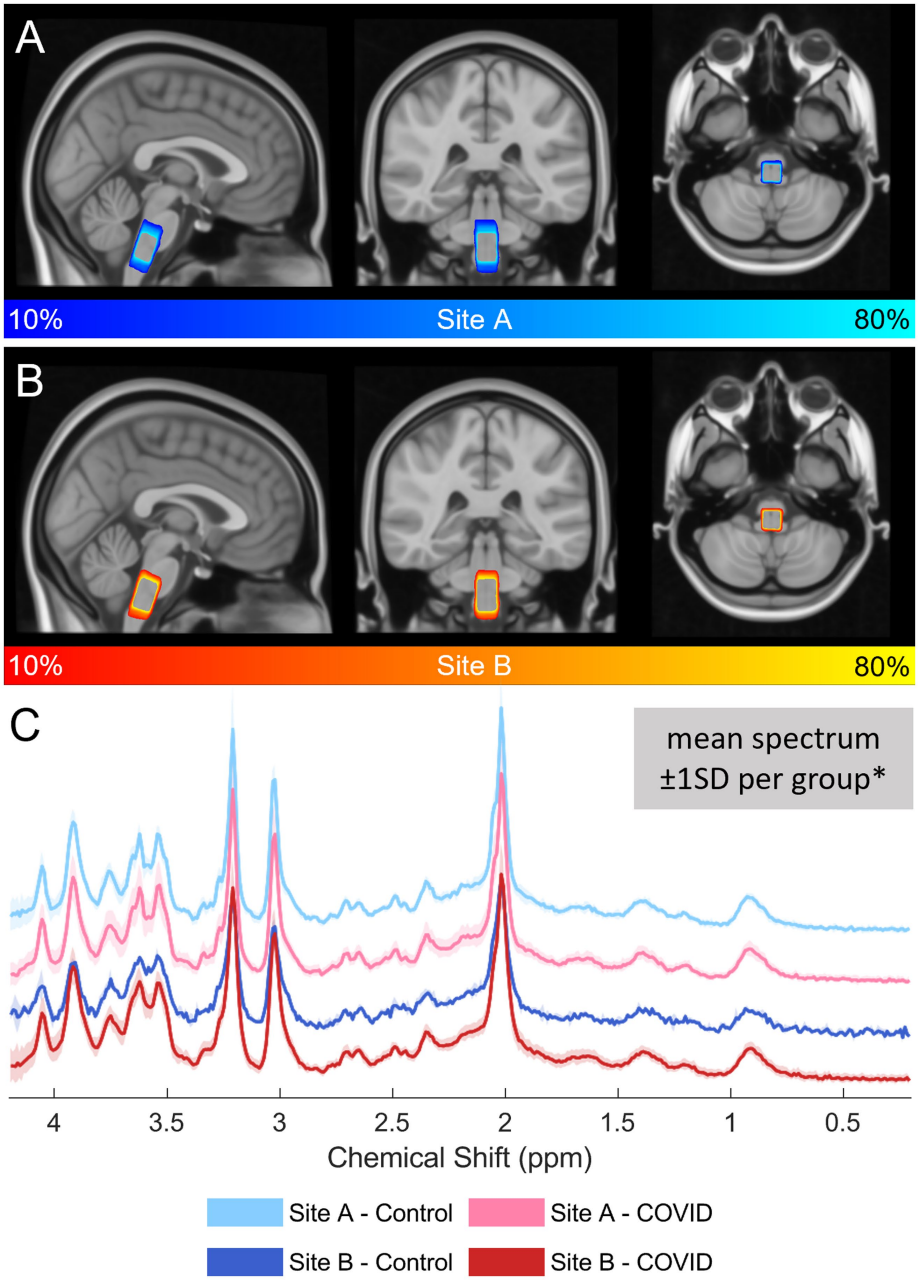
Visual quality assessment of sLASER data acquired in this study. **(A,B)** Precision of voxel placement at the ponto-medullary junction is assessed through heatmaps plotted in blue for Site A and orange for Site B. Voxel placement was consistent throughout the study, as evidenced by the CSF proportion between the sites (p(f_CSF_) = 0.064) and consistently high Sørensen–Dice coefficients (DSCs) of 75–77 (p(DSC) = 0.10). **(C)** Plots of mean spectra in each group. The shaded areas represent ±1 standard deviation. This shows consistent, high data quality. Quantitative measures of mean spectral quality were high—the signal-to-noise ratio (SNR) of N-acetyl aspartate (NAA) (SNR_NAA_) was 64.2 ± 17.7 (mean ± SD) and NAA’s linewidth measured by the full-width at half maximum (FWHM_NAA_) was 13.7 ± 2.6 Hz. SNR_NAA_ was similar between the sites (*p* = 0.33). FWHM_NAA_ was lower at Site A (difference = 16.1%, *p* = 0.0033). For detailed results, see Table 2 and Supplementary Figure 3. *Single subject spectra are included in Supplementary Figure 2. DSC, Sørensen–Dice coefficient; FWHM, full-width at half maximum; SNR, signal-to-noise ratio; sLASER, semi-adiabatic localization by adiabatic selective refocusing.

Voxel placement at the ponto-medullary junction was consistent, as shown in Figures 2A,B and indicated by comparable Sørensen–Dice coefficients (Equations 1 and 2) (difference = −2.6%, *p* = 0.10) and overall low CSF volume fractions (mean at both sites; f_CSF_ = 3.7%, Table 3). Spectral quality was ‘high’ [FWHM_NAA_ < the threshold defined by the MRS Expert Consensus rating scale (Juchem et al., 2021)], except for one spectrum, which was rated as ‘acceptable’. The participants’ individual processed spectra are shown in Supplementary Figure 2. The mean spectra, averaged across all participants within each group, are shown in Figure 2C. The mean metabolite linewidth was 13.7 Hz, and the mean metabolite signal-to-noise ratio was 64.2. The narrow linewidths led to good separation of glutamate (Glu) and glutamine (Gln) (r_min_(Glu, Gln) = −0.26). Further details are provided in Table 3 and presented in Supplementary Figures 3, 4.

**Table 3:** Spectroscopy analysis result summary.

Spectral quality metrics	Site A				Site B				Cross-site comparison			
	Control	Patients	Diff. (%)	*p*-value	Control	Patients	Diff. (%)	*P*-value	Site A	Site B	Diff. (%)	*P*-value
DSC	76.2 ± 4.1	74.7 ± 5.1	−2.0	0.47	78.1 ± 1.8	77.1 ± 3.1	−1.3	0.42	75.3 ± 4.7	77.3 ± 2.9	2.6	0.10
f_CSF_ (%)	4.1 ± 2.6	2.3 ± 1.5	−56.3	0.096	3.2 ± 1.6	4.7 ± 2.9	38.0	0.18	3.0 ± 2.2	4.4 ± 2.7	37.8	0.064
SNR_NAA_	60.1 ± 14.9	71.5 ± 22.8	17.3	0.17	67.5 ± 19.1	60.2 ± 13.7	−11.4	0.51	66.8 ± 20.4	61.5 ± 14.6	−8.3	0.33
FWHM_NAA_ (Hz)	12.9 ± 2.5	12.5 ± 2.2	−3.1	0.72	14.7 ± 0.9	14.9 ± 2.7	1.4	0.78	12.6 ± 2.3	14.8 ± 2.4	16.1	0.0033
Metabolite concentrations (mMol/L)												
tNAA	16.2 ± 0.6	16.8 ± 1.0	3.6	0.096	17.9 ± 1.1	16.8 ± 0.9	−6.3	0.14	16.6 ± 0.9	17.0 ± 1.0	2.4	0.16
tCr	11.6 ± 0.9	11.4 ± 0.6	−1.7	0.51	12.4 ± 0.7	12.3 ± 1.2	−0.8	0.69	11.5 ± 0.7	12.3 ± 1.1	6.7	0.0068
Ins	15.5 ± 1.4	16.2 ± 2.2	4.4	0.36	16.8 ± 3.0	16.4 ± 1.2	−2.4	0.77	15.9 ± 1.9	16.5 ± 1.6	3.7	0.33
Glu	7.2 ± 0.6	7.3 ± 0.7	1.4	0.78	8.3 ± 1.8	7.4 ± 0.9	−11.5	0.38	7.3 ± 0.7	7.6 ± 1.1	4.0	0.33
tCho	4.6 ± 0.7	4.7 ± 0.7	2.2	0.63	5.0 ± 0.8	4.9 ± 0.7	−2.0	0.81	4.7 ± 0.7	4.9 ± 0.7	4.2	0.27
Gln	0.06 ± 0.17	0.00 ± 0.00	−200.0	0.35	0.34 ± 0.68	0.19 ± 0.41	−56.6	0.70	0.02 ± 0.11	0.22 ± 0.45	166.7	0.062
GABA	0.87 ± 0.74	1.35 ± 0.59	43.2	0.13	1.10 ± 0.99	0.77 ± 0.73	−35.3	0.56	1.16 ± 0.68	0.83 ± 0.77	−33.2	0.14

Spectral quality metrics include signal-to-noise ratio (SNR) and full-width at half maximum (FWHM) linewidth of the main N-acetyl aspartate (NAA) peak. The fraction of CSF contribution and the Sørensen–Dice coefficients (DSC) for voxel position are measures of voxel placement accuracy and precision, respectively. Summary metrics are reported as mean ± SD and only include data after the exclusion of incomplete MRI data or poor-quality spectra. We report metabolite concentrations (mean ± SD) for each group at the two sites, as well as for all participants combined at each site (both patients and controls) in the cross-site comparison. Concentrations are referenced to the non-suppressed water signal and corrected for CSF contribution. Values relative to total creatine are shown for comparison in Supplementary Table 3. DSC, Sørensen–Dice coefficient; f_CSF_, volumetric fraction of cerebrospinal fluid; FWHM, full-width at half maximum; GABA, γ-Aminobutyric acid; Gln, glutamine; Glu, glutamate; Ins, *myo*-inositol; NAA, N-acetyl aspartate; SNR, signal-to-noise ratio; tCho, choline containing compounds; tCr, combined creatine and phosphocreatine.

### Cross-site comparison

**Figure 3:**
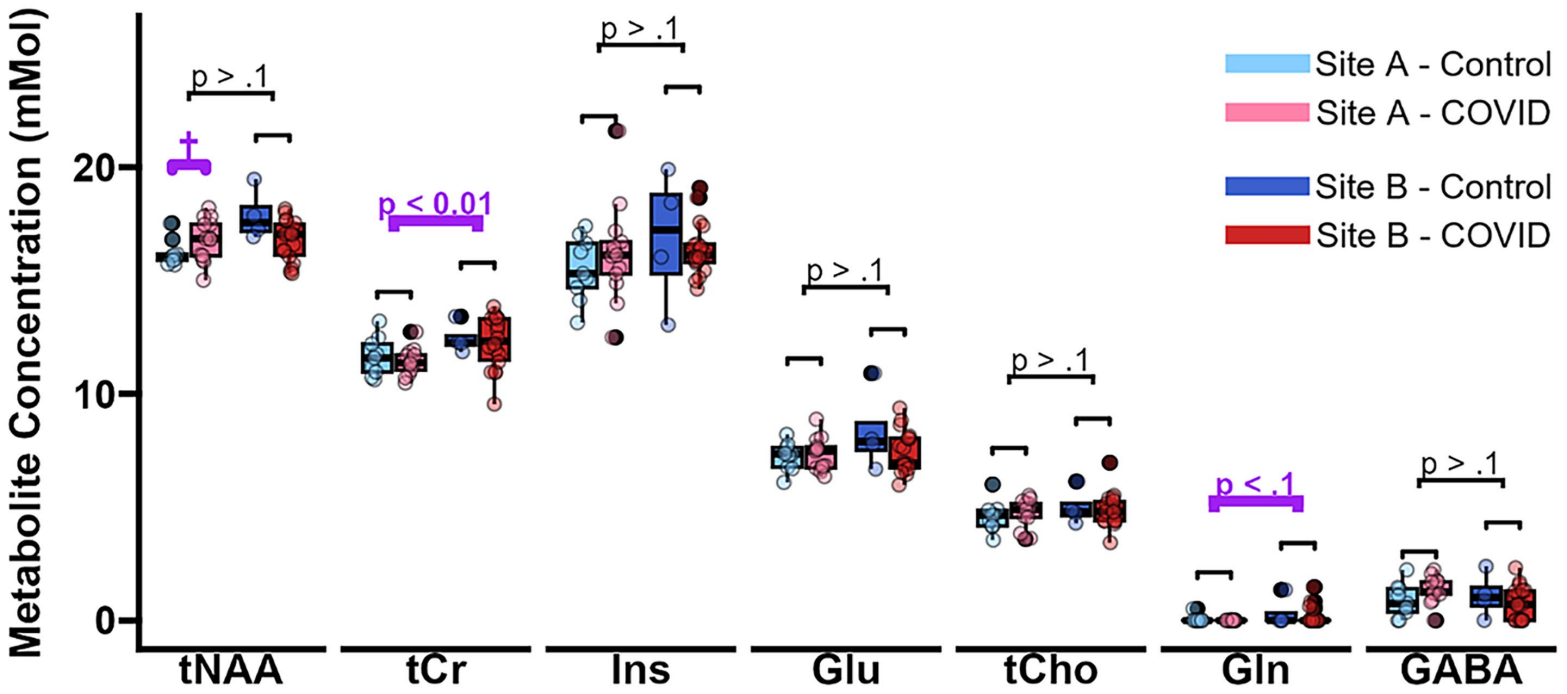
Metabolite concentrations corrected for tissue water content, reported for each group at both sites. Highlighted in purple are notable discrepancies between the sites, including a significant change in tCr (difference = 6.7%, *p* = 0.0068) and a trend for Gln (difference = 166.7%, *p* = 0.062). An equivalent plot normalised relative to tCr is provided for comparison (Supplementary Figure 5). Statistical comparisons were performed using two-sided t-tests. Brackets without labels indicate that the t-tests had a *p*-value > 0.1 (not significant, not a trend). GABA, *γ*-aminobutyric acid; Gln, glutamine; Glu, glutamate; Ins, *myo*-inositol; NAA, N-acetyl aspartate; tCho, choline containing compounds; tCr, combined creatine and phosphocreatine.

Water-referenced concentrations of N-acetyl aspartate, Ins, glutamate, choline and *γ*-Aminobutyric acid were consistent across the sites. However, water-referenced concentrations of total creatine were higher at Site B compared to Site A (12.3 vs. 11.5 mmol/L; 6.7%, *p* = 0.0068) (Figure 3; Table 3), and glutamine trended to be lower at Site A compared to Site B (0.02 vs. 0.22 mmol/L, 166.7%, *p* = 0.062). Concentrations referenced to combined creatine were more comparable across the sites (Supplementary Table 3; Supplementary Figure 5). Similar to concentrations corrected for CSF contribution, water-scaled concentrations corrected for cortical T_1_ relaxation losses were unable to remove the differences between the sites (Supplementary Figure 6).

### Effect of peak disease severity on metabolite concentrations

**Figure 4:**
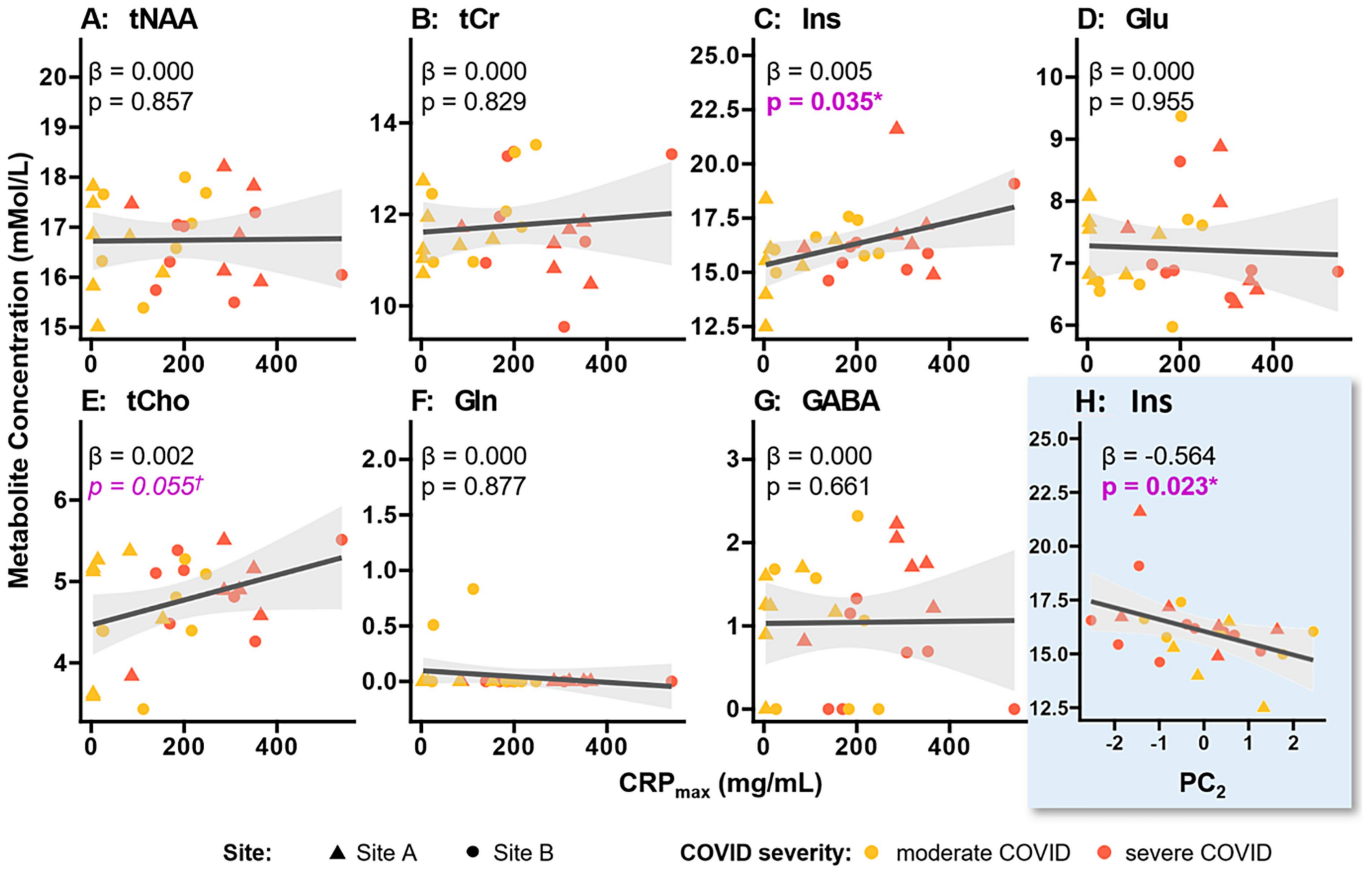
Correlation of clinical markers with metabolite concentrations. **(A–G)** The correlation between the highest C-reactive protein (CRP) assay value during hospital admission (CRP_max_) and metabolite concentration, as determined by the linear mixed model from Equation (3). Ins correlated positively with CRP_max_ (*p* = 0.035), which may reflect neuroinflammation; and tCho showed a positive trend (*p* = 0.055), which may reflect increased membrane turnover consistent with neuroinflammation. Modelling results are summarised in Table 4. **(H)** Correlation of the second principal component (PC_2_), which is highly loaded with patients’ mental health and anxiety outcomes (Supplementary Figures 10B,C). The negative correlation (*p* = 0.023) with Ins suggests that patients with overall poorer emotional wellbeing (lower PC_2_) have higher Ins levels. CRP, C-reactive protein; GABA, γ-aminobutyric acid; Gln, glutamine; Glu, glutamate; Ins, *myo*-inositol; NAA, N-acetyl aspartate; PC_2_, 2nd principal component; tCho, choline containing compounds; tCr, combined creatine and phosphocreatine.

We investigated the impact of the inflammatory response, as measured by the maximum CRP assay result during hospital admission (CRP_max_), in the patient group (Equation 3). Figure 4 shows a positive correlation between *myo*-inositol and CRP_max_ (*β* = 0.005; 95% CI 0.000 to 0.010; *p* = 0.035) in the patients. There was also a positive trend for total choline (*β* = 0.002; 95% CI 0.000 to 0.003, *p* = 0.055) in the patients (Table 4). These findings for water-scaled metabolite concentrations were consistent with those observed for creatine-referenced concentration ratios (Supplementary Table 4; Supplementary Figure 7).

**Table 4:** Results of linear mixed modelling for data from both sites, according to Equation (3).

Metabolite	Highest CRP during admission	
	Estimate, β (95% CI)	*p*-value
tNAA	0.000 (−0.002, 0.003)	0.857
tCr	0.000 (−0.003, 0.003)	0.829
Ins	0.005 (0.000, 0.010)	**0.035**
Glu	0.000 (−0.002, 0.002)	0.955
tCho	0.002 (0.000, 0.003)	0.055
Gln	0.000 (0.000, 0.000)	0.877
GABA	0.000 (−0.002, 0.003)	0.661

See Figure 4 for scatter plots. *p*-values below the significance threshold of 0.05 are highlighted in bold. CRP, C-reactive protein; GABA, γ-aminobutyric acid; Gln, glutamine; Glu, glutamate; Ins, *myo*-inositol; NAA, N-acetyl aspartate; tCho, choline containing compounds; tCr, combined creatine and phosphocreatine.

### Metabolic changes in the patients versus the controls

A linear mixed model was used to compare the patients’ and controls’ metabolite concentrations (Equation 4). This did not identify any significantly altered metabolism detectable by sLASER 7T MRS in the brainstem after correcting for age, sex and site effects (Supplementary Figures 8, 9).

### Impact of physical and mental wellbeing at follow-up

The first two components of physical and mental health accounted for 53 and 14% of the variance, respectively (Supplementary Figure 10A). The third and lower components each accounted for less than 10% of the total variance. The first component was loaded consistently across all clinical scores (mean cos^2^ ± sd = 53% ± 8%), representing a balanced combination of physical and mental wellbeing components. On the other hand, the second principal component presented a more heterogeneous symptom profile (mean cos^2^ ± sd = 14% ± 10%), where it was more impacted by components related to mental health (SF-36 Mental Health subscore, GAD-7 and PHQ-9). Itemised loading of the first two principal components is summarised in Supplementary Figures 10B,C.

The first principal component did not correlate with any of the measured metabolite concentrations (Equation 5, *p* > 0.19, Supplementary Table 5; Supplementary Figure 11), but the second component correlated with measured levels of Ins (*β* = −0.56, *p* = 0.023) (Figure 4H; Supplementary Table 5; Supplementary Figure 12).

## Discussion and Conclusion

It is physiologically plausible to hypothesise that neuroinflammation may contribute to persistent symptoms in individuals previously hospitalised with moderate-to-severe COVID-19, as we recently reported using quantitative susceptibility mapping (QSM) in this patient cohort (Rua et al., 2024). This aligns with other neuropathological studies of severe and fatal COVID-19 cases, which have identified SARS-CoV-2 viral material in brainstem nuclei, supporting the possibility of direct viral neuroinvasion (Matschke et al., 2020; Schwabenland et al., 2024; Emmi et al., 2023). We therefore measured brainstem metabolite concentrations in this group and in healthy controls. Spectral quality was high enough to allow reliable quantification of seven metabolites, indicating the feasibility of measuring *brainstem* metabolism at 7T in a clinical cohort across multiple sites. Whilst no significant differences were detected between the patients and controls, a measure of peak disease severity (maximum C-reactive protein assay value recorded during hospital admission) was correlated with *myo*-inositol concentration (a marker of glial activation) and showed a trend towards association with concentrations of choline-containing compounds (a potential marker of inflammation) in the brainstem. An exploratory analysis of clinical symptoms at follow-up suggested that patients with poorer mental health may have higher brainstem *myo*-inositol concentrations. Together, these findings suggest that there are enduring changes in brainstem neurochemistry in patients recovering from moderate-to-severe COVID-19, potentially linked to ongoing inflammatory changes in the brainstem. These findings align with imaging results from the same patient cohort using quantitative susceptibility mapping (QSM) in the brainstem (Rua et al., 2024). Nevertheless, QSM and MRS are sensitive to different biological substrates; QSM-detectable changes may reflect microstructural alterations or iron content but lack the biochemical specificity of MRS.

### Effects of peak disease severity

There was a significant positive correlation between *myo*-inositol concentration and inflammatory blood markers (*p* = 0.035) and a positive trend for total choline (*p* = 0.057) after correcting for differences in site, age and sex, as shown in Figure 4 and Table 4. We used the highest CRP assay result during hospital admission as an indicator of peak disease severity, instead of the World Health Organization severity scale, due to the latter’s restricted dynamic range of disease severity—from 3 (hospitalised, but no oxygen therapy administered) to 7 (mechanical ventilation and additional organ support required).

Myo-inositol is a glial cell marker. During neuroinflammation, activated glial cells, particularly astrocytes, release inflammatory mediators, as part of the brain’s immune response. This can lead to increased Ins concentration (Liddelow et al., 2017). This may explain why the patients with more severe illness had higher *myo*-inositol concentrations during scanning.

Furthermore, the slight increase in total choline may also provide evidence of an inflammatory response, since elevated choline levels are associated with increased membrane turnover and have previously been shown to be elevated in cases of severe brainstem encephalitis (Sa et al., 2018). This is also consistent with previous reports from animal models of neuroinflammation and other human neurodegenerative diseases, where it is said to reflect local glial activation in neuroinflammation (Genovese et al., 2021; Chaney et al., 2019).

### Overall metabolic changes in post-acute COVID-19

No differences in metabolite concentrations were detected between the patients and the controls. One possible explanation for this is the variability in timing of MRI assessments following initial hospital discharge, which could have impacted the yield or sensitivity of MRI measures for detecting group differences in brainstem neurochemistry.

Another possibility is that the absence of differences in metabolite concentrations could be masked by changes in either metabolite relaxation times (T_1_ or T_2_) or tissue water content. These changes could, for example, be due to oedema associated with neuroinflammation. We did not measure metabolite T_1_ or T_2_ values or tissue water content in this study due to scan time limitations aimed at ensuring patient comfort, especially given that scans were acquired under exceptional conditions during the acute COVID-19 lockdowns. Although we did not measure metabolite T_2_ values directly, we can infer from the similar linewidths of the metabolite peaks in the patients and controls that there were no substantial changes in metabolite T_2_ values.

### Impact of physical and mental wellbeing at follow-up

Symptom profiles at follow-up showed a correlation between the second principal component, which was highly loaded with mental health-related outcomes, and *myo*-inositol (*p* = 0.035). Higher *myo*-inositol levels were associated with poorer mental wellbeing at follow-up.

We note that *myo*-inositol has previously been shown to *decrease* with poorer mental wellbeing in clinical depression and anxiety (Concerto et al., 2023). This difference likely reflects differences in the pathophysiology of acute infection (COVID-19) compared to major depressive disorders.

The observed correlations between increased *myo*-inositol, systemic inflammation (maximum CRP value) and poorer mental wellbeing support our hypothesis that brainstem neuroinflammation occurs in COVID-19 and may persist for months after the initial infection, as reflected by symptoms at follow-up. These observations are consistent with a role for brainstem neuroinflammation during the post-acute phase of COVID-19.

### Limitations

This study commenced during the initial phase of the COVID-19 pandemic. Consequently, the intervals between hospital discharge, outpatient follow-up assessments and 7T MRI varied, as data were acquired from patients who were admitted between March 2020 and February 2021, covering multiple lockdowns in the UK. Our study cohort included no patients or controls who had received immunisations; consequently, patients often had clinically severe COVID-19. Recruitment focused on individuals who were hospitalised for COVID-19 and neurologically healthy controls who had never tested positive for COVID-19 and had no prior history of COVID-19 symptoms. Therefore, CRP was not measured in this control cohort. Quality of life metrics (SF-36, PHQ-9, GAD-7) were only assessed during the outpatient follow-up visit prior to the 7T MRI assessment. Hence, it is not possible to be certain that the symptoms did not precede the COVID-19 episode. At this time, recruiting additional control subjects is unlikely because the endemic status of COVID-19 and the widespread uptake of vaccination against COVID-19 mean that there are now few people who have never had COVID-19 infection and who do not have COVID-19 antibodies due to vaccination.

The relatively small sample sizes within each subgroup (controls and patients at each site), ranging between 5 and 20 participants, caused challenges in detecting subtle changes in metabolite concentrations due to reduced statistical power. We were not able to perform serum tests on the controls and instead relied on their reported lack of history of COVID-19 infection.

A limitation of this study is the difference in some of the metabolite concentrations between Site A and Site B, as shown in Figure 3. We believe that these differences are due to technical features of the first-generation Magnetom 7T MRI platform at Site B, which led to variable TR. The apparent between-site differences may be attributable to limitations in the harmonisation of the acquisition protocol across scanner platforms, which unfortunately cannot be corrected retrospectively. This is especially apparent since combined creatine and glutamine each have relatively long T_1_s and are therefore more impacted by variations in repetition time. Concentrations referenced to combined creatine were more comparable across the sites (Supplementary Table 3; Supplementary Figure 5); however, care should be taken when interpreting these values since we found evidence of changes in tCr between the groups. Equally, water-scaled concentrations corrected for cortical T_1_ relaxation losses were unable to remove the differences between the sites (Supplementary Figure 6). Future studies should prioritise maintaining a fixed (slightly longer) TR to facilitate robust comparisons of concentrations of metabolites whose T_1_ relaxation time is uncertain.

Motivated by a working theory that neuropsychiatric and respiratory symptoms reported in patients who have been hospitalised with COVID-19 may be due to persisting abnormalities in key neuromodulatory nuclei and respiratory control centres located in the brainstem near the ponto-medullary junction, we used the smallest spectroscopy voxel size that provided a good signal-to-noise ratio in pilot scans in volunteers. However, the 2.88 cm^3^ voxel volume that we selected is several orders of magnitude larger than the individual components of the brainstem (Powell, 2006), which is a complex structure composed of tightly packed small nuclei and tracts including those involved in cardiovascular and respiratory control (Romano et al., 2019). This limits the spatial specificity of spectroscopy and precludes confident attribution of metabolite changes to individual nuclei. In addition, many (perhaps even the majority) of COVID-19 cases have no direct CNS infection—although the true frequency is unknown. Indeed, the improvements in outcomes with corticosteroids and IL-6 antagonists in clinical trials suggest that even extracranial pathology is likely to be primarily driven by an excessive and maladaptive host response. However, parcellating these two mechanisms in individual patients is not straightforward. This was the reason we selected CRP as a means of quantifying illness severity, as it integrates both the viral infection and the host response. Similarly, the cause and mechanistic basis of post-COVID-19 symptoms are complex. We do not claim that the brainstem changes we found are the sole cause of the symptoms, but rather that they are a plausible mechanistic driver.

Since the brainstem is surrounded by CSF, we employed high-bandwidth GOIA-WURST pulses to minimise chemical shift displacement errors (Clarke et al., 2020).

Therefore, although it is an attractive hypothesis, this study cannot say whether COVID-19 causes neuroinflammation in the brainstem respiratory centres.

### Conclusion

The patients’ brainstem *myo*-inositol concentration correlated positively with inflammatory markers during hospital admission and showed a trending correlation with total choline. At follow-up, poorer mental health was associated with higher brainstem *myo*-inositol concentrations. This supports a possible link between COVID-19, brainstem neuroinflammation and ongoing symptoms. This study also shows that sLASER 7T MRS of the *brainstem* is feasible in patients who were hospitalised with COVID-19 and in healthy volunteers within a multi-site study. Spectral quality was rated as good or excellent in 90% of all participants.

## Data Availability

The datasets presented in this article are not readily available because of restrictions imposed by our institutions on privacy/ethical grounds. Requests to access the datasets should be directed to the corresponding author.

## Group members of the Cambridge NeuroCOVID

Kieren Allinson, Fahim Anwar, Junaid Bhatti, Edward T Bullmore, Doris A Chatfield, David Christmas, Alasdair J Coles, Jonathan P Coles, Marta Correia, Tilak Das, Anne Elmer, Paul C Fletcher, Alasdair W Jubb, Victoria C Lupson, Anne Manktelow, David K Menon, Andrew Michell, Edward Needham, Virginia FJ Newcombe, Joanne G Outtrim, Linda Pointon, Christopher T Rodgers, James B Rowe, Catarina Rua, Stephen J Sawcer, Nyarie Sithole, Lennart RB Spindler, Emmanuel A Stamatakis, Jonathon Taylor, Fernanda Valerio, Barry Widmer, Guy B Williams.

## Group members of the CITIID-NIHR COVID-19 BioResource Collaboration

John Allison, Gisele Alvio, Stephen Baker, Sharon Baker, Laura Bergamashi, Nonantzin Beristain-Covarrubias, Areti Bermperi, Ariana Betancourt, Heather Biggs, Lucy Booth, Rebecca Boston, Georgie Bower, John Bradley, Karen Brookes, Ashlea Bucke, Ben Bullman, Helen Butcher, Sarah L. Caddy, Jo Calder, Laura G. Caller, Laura Canna, Daniela Caputo, Matt Chandler, Yasmin Chaudhry, Patrick Chinnery, Debbie Clapham-Riley, Daniel Cooper, Chiara Cossetti, Cherry Crucusio, Isabel Cruz, Martin D. Curran, Ranalie de Jesus, Aloka De Sa, Katie Dempsey, Eleanor Dewhurst, Giovanni Di Stephano, Jason Domingo, Gordon Dougan, Benjamin J. Dunmore, Anne Elmer, Maddie Epping, Stuart Fawke, Theresa Feltwell, Christian Fernandez, Alexander Ferreira, Stewart Fuller, Anita Furlong, Iliana Georgana, Ian Goodfellow, Stefan Gräf, Barbara Graves, Jennifer Gray, Richard Grenfell, Ravindra Gupta, Thevinya Gurugama, Lihinya Gurugama, Grant Hall, William L. Hamilton, Julie Harris, Sabine Hein, Sarah Hewitt, Andrew Hinch, Josh Hodgson, Emily C. Horner, Myra Hosmillo, Zhaleh Hosseini, Charlotte J. Houldcroft, Christopher Huang, Robert Hughes, Oisin Huhn, Kelvin Hunter, Tasmin Ivers, Rhys Izuagbe, Aminu S. Jahun, Isobel Jarvis, Heather Jones, Emma Jones, Sherly Jose, Maša Josipović, Mary Kasanicki, Jane Kennet, Fahad A. Khokhar, Yvonne King, Rebecca King, Nathalie Kingston, Jenny Kourampa, Anna G. Kovalenko, Emma Le Gresley, Ekaterina Legchenko, Paul J. Lehner, Daniel Lewis, Emily Li, Rachel Linger, Paul A. Lyons, Joe Marsden, Jennifer Martin, Nicholas J. Matheson, Caroline McMahon, Anne Meadows, Sarah Meloy, Vivien Mendoza, Luke W. Meredith, Federica Mescia, Alexei Moulton, Francesca Muldoon, Thomas Mulroney, Criona O’Brien, Ciara O’Donnell, Charmain Ocaya, Ommar Omarjee, Nigel Ovington, Sofia Papadia, Roxana Paraschiv, Surendra Parmar, Ciro Pasquale, Christopher J. Penkett, Marlyn Perales, Marianne Perera, Isabel Phelan, Malte L. Pinckert, Linda Pointon, Petra Polgarova, Nicole Pond, Jane Price, Venkatesh Ranganath, Rebecca Rastall, Carla Ribeiro, Nathan Richoz, Nika Romashova, Jane Rowlands, Valentina Ruffolo, Maria Rust, Abigail Sage, Jennifer Sambrook, Caroline Saunders, Natalia Savoinykh, Ingrid Scholtes, Katherine Schon, Mayurun Selvan, Rahul Sharma, Joy Shih, Kenneth G. Smith, Sarah Spencer, Hannah Stark, Kathleen E. Stirrups, Mateusz Strezlecki, Charlotte Summers, Rachel Sutcliffe, James Thaventhiran, Tobias Tilly, Zhen Tong, Hugo Tordesillas, M. Estee Torok, Mark R. Toshner, Paul Townsend, Carmen Treacy, Lori Turner, Phoebe Vargas, Bensi Vergese, Neil Walker, Laura Watson, Jennifer Webster, Michael P. Weekes, Kate Williamson, Jennifer Wood, Jieniean Worsley, Marta Wylot, Anna Yakovleva, Juan Carlos Yam-Puc, Julie-Ann Zerrudo.
