# Supplementary material for: Brainstem neurochemical profiles after hospitalisation for COVID-19: a 7T MR spectroscopy study
Source: Front Neurosci. 2025 Oct 15;19:1617709. doi: 10.3389/fnins.2025.1617709 (PMC12568683; doi:10.3389/fnins.2025.1617709)
Supplement: Supplementary file 1 [file Data_Sheet_1.DOCX]

**Supplementary Material**

To accompany “Brainstem neurochemical profiles after hospitalisation for COVID-19: a 7T MR spectroscopy study”.

Table of Contents

Supplementary Methods 4

Note on Metropolis Hastings fitting on FSL-MRS 4

Supplementary Exploratory Cluster Analysis 5

Supplementary Tables 6

Supplementary Table 1 6

Supplementary Table 2 7

Supplementary Table 3 10

Supplementary Table 4 11

Supplementary Table 5 12

Supplementary Figures 13

Supplementary Figure 1 13

Supplementary Figure 2 14

Supplementary Figure 3 15

Supplementary Figure 4 15

Supplementary Figure 5 17

Supplementary Figure 6 18

Supplementary Figure 7 19

Supplementary Figure 8 20

Supplementary Figure 9 21

Supplementary Figure 10 22

Supplementary Figure 11 23

Supplementary Figure 12 24

Supplementary Figure 13 25

Supplementary Figure 14 26

Supplementary Figure 15 27

Supplementary Figure 16 28

Supplementary References 29

## Supplementary Methods

### **Note on Metropolis Hastings fitting on FSL-MRS**

Spectra were fitted using the Metropolis Hastings (MH) algorithm [1, 2]. This fits a linear combination of basis spectra in the frequency domain to model each metabolite and a second order polynomial baseline that models macromolecule signals. We used a basis set simulated for this specific sequence and a measured macromolecular baseline [3]. The MH fit was initialised with a conventional truncated Newton fit. Then MH sampling was performed with 100 burn-in steps and 10,000 sampling steps per subject.

MH fitting gives a probability distribution for each fitted parameter. The distributions for each metabolite signal amplitude were summarised by fitting them to folded-normal distributions (defined according to their mean μ and standard deviation σ) using foldnorm.mle from the Rfast library [4] (version 2.0.8, 2023) in R [5] (v4.3.1; R Core Team, 2023). This is appropriate because metabolite concentrations are by definition non-negative real numbers, and hence so are the unknown true noiseless metabolite signal amplitudes. Using the folded-normal distribution mitigates against noise rectification for small signals. For each metabolite in each scan, subsequent statistical analysis was based on the fitted folded-normal mean amplitude μ as a best estimate of the metabolite signal amplitude, and the fitted folded-normal standard deviation of the amplitude σ as an estimate of the uncertainty in determining the mean amplitude.

## Supplementary Exploratory Cluster Analysis

**Methods**: We conducted an additional exploratory analysis using k-means clustering on the main metabolite concentrations of all 44 spectra with acceptable or high spectral quality. Fisher’s Exact Test and unpaired, two-sided t-tests were used for further exploratory analysis. Raw, uncorrected p-values are reported.

**Results**: We were able to identify 2 “weak” clusters (mean silhouette width of 0.307) using the silhouette method (**Supplementary** **Figure 13B**) [6]. The two clusters were linearly separable by the first principal component of the metabolite profile (**Supplementary** **Figure 14**). The clusters did not discriminate between the two sites (**Supplementary** **Figure 14A**, p(Fisher’s Exact Test) = 0.20) which shows that for cluster analysis the TR variation discussed in the main manuscript did not have a critical impact. *Group* (i.e. patient or control) was also not a significant discriminator between Cluster 1 and 2 (p = 0.49).

However, we observed that the majority of controls (N = 10) was assigned to Cluster 2 (**Supplementary** **Figure 14B**) with only N=3 in Cluster 1 whereas the patients are more divided (N=19 in cluster 2 vs N=12 in cluster 1). We therefore tentatively interpret metabolite profiles in Cluster 2 as representing “more normal” individuals. To explore what may distinguish ‘more normal’ patients in cluster 2 from the presumably ‘less normal’ patients in cluster 1 we compared metabolites for patients in the two clusters. All of Glu, Ins, tCho, tCr and tNAA were significantly higher for patients in Cluster 1 (**Supplementary** **Figure 15**). **Supplementary** **Figure 16** shows boxplots of key metrics from the patient only population. Patients in Cluster 1, had significantly lower emotional wellbeing scores (PC_2_) at their outpatient follow-up visits (p(t-test)=0.0094), and a trend (p=0.064) to higher maximum CRP values during their hospitalisation for acute COVID-19.

**Discussion**/**Conclusion**: Overall, these findings suggest that patients whose metabolite profiles resembled controls, had better mental wellbeing in the long-term and showed a trend to having had less severe systemic inflammatory responses during their acute disease phase. However, we recommend caution in interpreting these findings since the strength of clustering was “weak” (mean silhouette width of 0.307).

## Supplementary Tables

### Supplementary Table 1

**Supplementary Table 1:** MP2RAGE sequence details. These were harmonised in the UK7T project [7].

| **Resolution (mm)** | - 1. x 0.7 x 0.7 |
| --- | --- |
| **Matrix size** | 224 x 224 x 224 |
| **Echo Time, TE (ms)** | 2.64 |
| **Repetition Time, TR (ms)** | 3500 |
| **Bandwidth per Pixel (Hz)** | 300 |
| **Inversion Times, TIs (ms)** | 725 and 2150 |
| **Acquisition Time (m:ss)** | 7:51 |

### Supplementary Table 2

**Supplementary Table 2:** MRS study details recommended by the recent MRS Experts Consensus paper [8].

| **Site Name** | **Site A** | **Site B** |
| --- | --- | --- |
| 1. Hardware |  |  |
| a. Field strength [T] | 7 T | |
| b. Manufacturer | Siemens Healthineers, Erlangen, Germany | |
| c. Model (software version if available) | Magnetom TERRA 7T (VE12) | Magnetom 7T (VB17) |
| d. RF coils: nuclei (transmit/receive), number of channels, type, body part | 1Tx/32Rx 1H Head Coil, Nova Medical | |
| e. Additional hardware | N/A | |
| 2. Acquisition |  |  |
| a. Pulse sequence | sLASER (CMRR package) | |
| b. Volume of interest (VOI) and its locations | Ponto-medullar junction in brainstem | |
| c. Nominal VOI size [cm^3^] | 12x12x20mm³ = 2.88 cm^3^ | |
| d. Repetition Time (TR), Echo Time (TE) [s, ms] | TR = 5 s TE = 28 ms | TR = 5.00s - 7.69s, mean 6.44s TE = 28ms |
| e. Total number of excitations or acquisitions per spectrum (NA)  Total number of spectra (acquired / in time-series) | *120 acquisitions per spectrum (NA = 120)* | |
| f. Additional sequence parameters (spectral width in Hz, number of spectral points, frequency offsets) | - N (spectral points) = 2048, - dwell time = 0.167ms - bandwidth = 6.00 kHz - frequency offset for metabolite excitations = -2.0ppm | |
| g. Water suppression method | - VAPOR - Water suppression Bandwidth = 135Hz | |
| h. Shimming method, reference peak, and thresholds for “acceptance of shim” chosen | FASTMAP applied to water signal  We aimed for <15Hz linewidth of unsuppressed water peak at the time of scan. Due to the one-off opportunity to scan patients, we did not abort scans or rescan if this was not achieved. | |
| i. Triggering or motion correction method | None | |

| 3. Data analysis methods and outputs |  |  |
| --- | --- | --- |
| a. Analysis software | FSL-MRS v2.1.12 | |
| b. Processing steps deviating from quoted reference or product analysis software (vendor, version) | none | |
| c. Output measure  (e.g. absolute concentration, institutional units, ratio) Processing steps deviating from quoted reference or product | 1. Fitted metabolite signal amplitudes, *A_met_*, were scaled using the unsuppressed water signal, A_water ref_, corrected for the volumetric fraction of cerebrospinal fluid (CSF) in the voxel, *f_CSF_,* and scaled by a global scaling factor, f_scale,global_. Concentrations are reported in mM.   \| $[met]$ \| $=\frac{1}{1-f_{\mathrm{CSF}}}\cdot f_{scale,global}\cdot\frac{A_{\mathrm{met}}}{A_{water ref}}$ \| Eq. (1) \| \| --- \| --- \| --- \|   Where  $f_{scale,global}=WCONC\times WVIS$  With the constants:   - Pure water concentration: WCONC = 55500 mmol/L [9] - Water visibility in WM: WVIS = 0.65 [9]   2. We also report fitted metabolite signal amplitudes scaled relative to total creatine (tCr):  $\frac{\left[ \mathrm{met} \right]}{\left[ \mathrm{tCr} \right]}=\frac{A_{met}}{A_{tCr}}$ | |
| d. Quantification references and assumptions, fitting model assumptions | - Pure water concentration: WCONC = 55500 mmol/L [9] - Water visibility in WM: WVIS = 0.65 [9]   The CMRR-provided basis set contained 20 simulated metabolite contributions and an experimentally measured macromolecular baseline.  Model fitting was initialised using the Truncated Newton algorithm, followed by the Metropolis Hastings (MH) algorithm with 10,000 iterations per subject. The MH fitted signal amplitude distributions were then summarised by fitting to a folded-normal distribution in R (v4.3.1; R Core Team, 2023) as implemented in the foldnorm.mle function of the Rfast library (version 2.0.8, 2023) to give a mean signal amplitude μ and standard deviation in the signal amplitude σ, which we took as a measure of uncertainty in the mean signal amplitude.  Our scripts for processing and fitting and our basis set are available on request. | |
| 4. Data Quality |  |  |
| a. Reported variables  (SNR, Linewidth (with reference peaks)) | NAA SNR  NAA FWHM linewidth | |
| b. Data exclusion criteria | Any transients deviating by a standard deviation of more than 2.58 were excluded before averaging. Additionally, spectra showing ghosting, lipid or motion artefacts during visual inspection were excluded from further analysis. | |
| c. Quality measures of postprocessing Model fitting (e.g. CRLB, goodness of fit, SD of residual) | - NAA SNR ≥ 35 - NAA linewidth < 20 Hz - Water linewidth < 13 Hz | |
| d. Sample Spectrum | **Figure 2C** & **Supplementary Figure 2** | |

### Supplementary Table 3

**Supplementary Table 3:** Extension to main results Table 3 with metabolite ratios reported as mean ± 1sd, percent difference (% diff.) and the p-value of a two-sample t-test. All p-values are reported as raw and uncorrected. *Italic* entries indicate trends with 0.05 < p < 0.1.

|  | **Site A** | | | | **Site B** | | | | **Cross-site Comparison** | | | |
| --- | --- | --- | --- | --- | --- | --- | --- | --- | --- | --- | --- | --- |
| **Concentration Ratios** | **Control** | **Patients** | **% diff.** | **p** | **Control** | **Patients** | **% diff.** | **p** | **Site A** | **Site B** | **% diff.** | **p** |
| tNAA/tCr | 1.40 ± 0.12 | 1.48 ± 0.12 | 5.6 | 0.16 | 1.44 ± 0.04 | 1.38 ± 0.12 | -4.3 | *0.09* | 1.45 ± 0.12 | 1.39 ± 0.11 | -4.2 | 0.11 |
| Ins/tCr | 1.34 ± 0.13 | 1.42 ± 0.19 | 5.8 | 0.22 | 1.35 ± 0.22 | 1.34 ± 0.10 | -0.7 | 0.93 | 1.39 ± 0.17 | 1.34 ± 0.13 | -3.7 | 0.33 |
| tCho/tCr | 0.62 ± 0.06 | 0.64 ± 0.08 | 3.2 | 0.51 | 0.67 ± 0.11 | 0.60 ± 0.06 | -11.0 | 0.35 | 0.64 ± 0.07 | 0.62 ± 0.07 | -3.2 | 0.36 |
| Glu/tCr | 0.40 ± 0.06 | 0.42 ± 0.06 | 4.9 | 0.49 | 0.40 ± 0.04 | 0.40 ± 0.05 | 0.0 | 0.96 | 0.41 ± 0.06 | 0.40 ± 0.05 | -2.5 | 0.63 |
| Gln/tCr | 0.00 ± 0.01 | 0.00 ± 0.00 | n.a. | 0.35 | 0.03 ± 0.05 | 0.02 ± 0.03 | -40.0 | 0.73 | 0.00 ± 0.01 | 0.02 ± 0.03 | 200.0 | *0.06* |
| GABA/tCr | 0.08 ± 0.06 | 0.12 ± 0.05 | 40.0 | 0.13 | 0.09 ± 0.07 | 0.06 ± 0.06 | -40.0 | 0.56 | 0.10 ± 0.06 | 0.07 ± 0.06 | -35.3 | *0.06* |

### Supplementary Table 4

Supplementary Table 4: Results from linear mixed modelling for creatine referenced concentrations.

Data from both sites were fitted with a linear model as described in Eq (3). See Supplementary Figure 6 for scatter plots.

|  | Highest CRP During Admission | |
| --- | --- | --- |
|  | Estimate, β (95% CI) | *p* Value |
| **tNAA/tCr** | 3.6E-05 (-3.2E-04, 3.9E-04) | 0.835 |
| **Ins/tCr** | 4.3E-04 ( 4.1E-05, 8.3E-04) | **0.032** |
| **Glu/tCr** | -1.2E-05 (-1.6E-04, 1.4E-04) | 0.867 |
| **tCho/tCr** | 1.3E-04 (-9.8E-06, 2.7E-04) | *0.067* |
| **Gln/tCr** | -2.2E-06 (-3.1E-05, 2.7E-05) | 0.877 |
| **GABA/tCr** | 5.5E-05 (-1.2E-04, 2.3E-04) | 0.510 |

### Supplementary Table 5

Supplementary Table 5: Extension to linear mixed modelling results shown in Table 4

*for data from both sites, according to Eq (3). See Supplementary Figures 10 and 11 for scatter plots*.

|  | **PC_1_** | | **PC_2_** | |
| --- | --- | --- | --- | --- |
|  | Estimate, β (95% CI) | *p* Value | Estimate, β (95% CI) | *p* Value |
| **tNAA** | 0.080 (-0.083, 0.242) | 0.319 | -0.043 (-0.366, 0.280) | 0.785 |
| **tCr** | -0.068 (-0.230, 0.094) | 0.390 | -0.160 (-0.472, 0.152) | 0.297 |
| **Ins** | -0.012 (-0.296, 0.273) | 0.933 | -0.564 (-1.043, -0.086) | **0.023** |
| **Glu** | 0.051 (-0.085, 0.187) | 0.447 | -0.178 (-0.439, 0.082) | 0.168 |
| **tCho** | -0.063 (-0.158, 0.032) | 0.183 | -0.130 (-0.310, 0.049) | 0.146 |
| **Gln** | -0.006 (-0.035, 0.023) | 0.655 | -0.016 (-0.053, 0.021) | 0.383 |
| **GABA** | 0.023 (-0.088, 0.135) | 0.666 | -0.095 (-0.312, 0.122) | 0.372 |

## Supplementary Figures

### Supplementary Figure 1


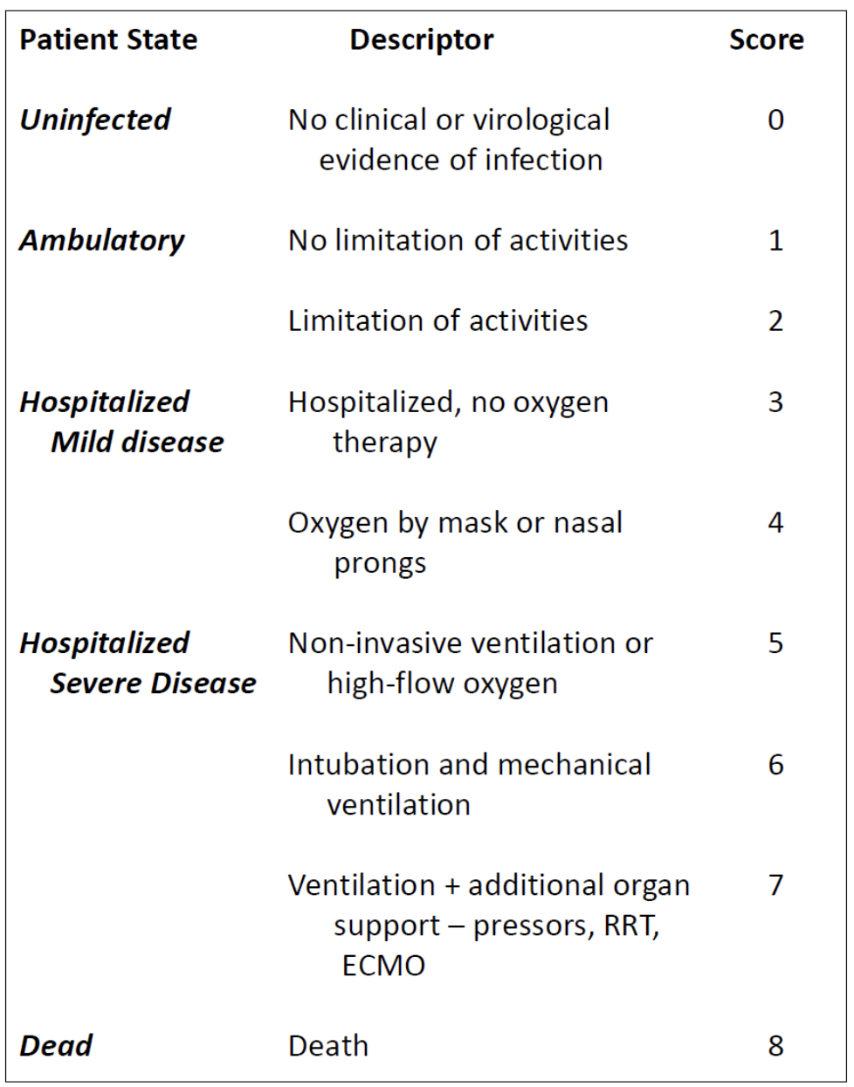


**Supplementary Figure 1:** World Health Organisation Ordinal Scale for Clinical Improvement [10].
From: World Health Organization WHO R&D Blueprint: Novel Coronavirus COVID-19 Therapeutic Trial Synopsis. 2020.

### Supplementary Figure 2


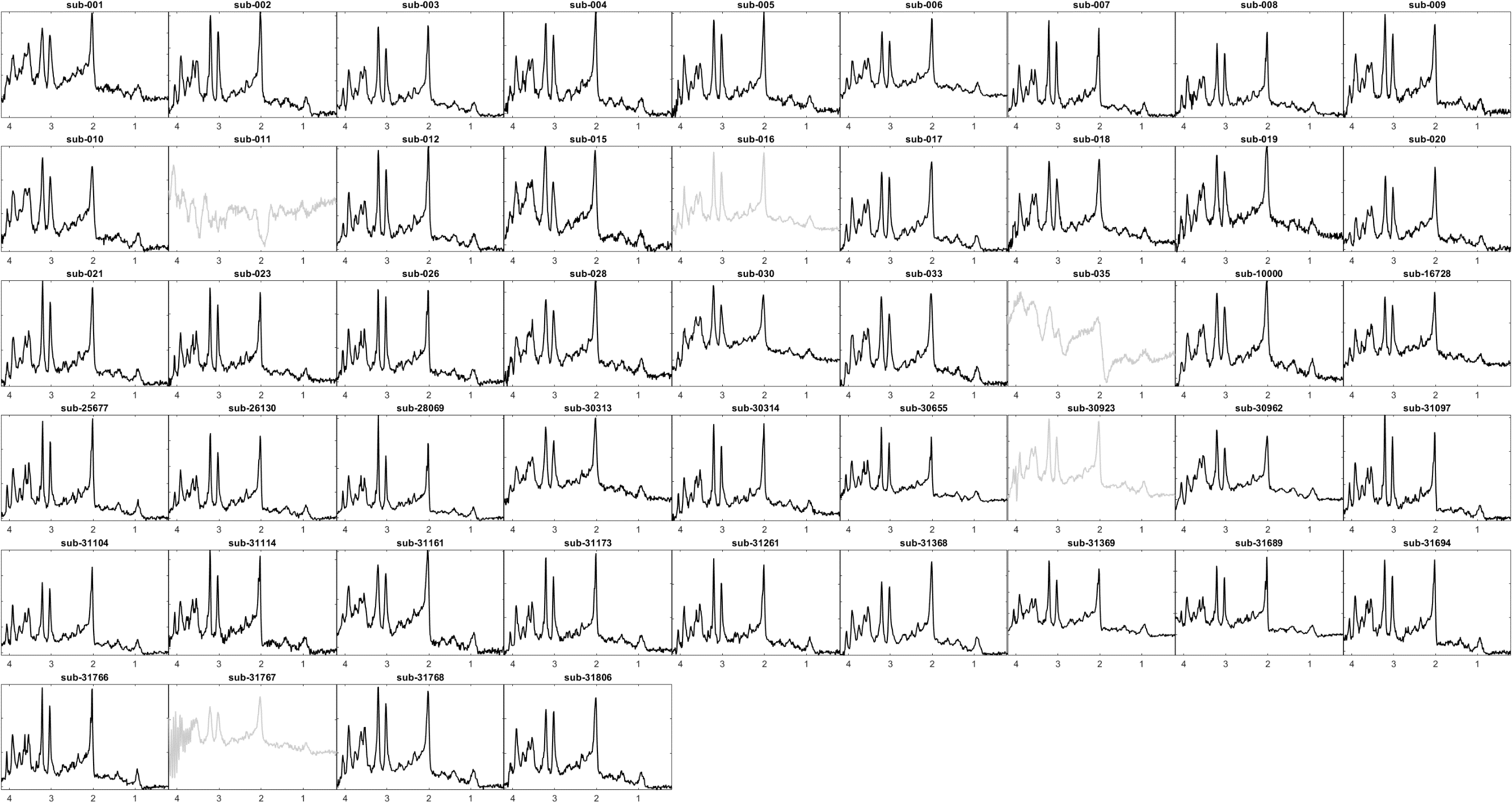


**Supplementary Figure 2:** Individual Spectra for each subject following eddy current correction, frequency alignment, phase correction and averaging. Spectra in grey did not pass visual QA (sub-011, sub-035, sub-31767), were excluded because of a positive COVID-19 anti-body test (sub-016) or due to an unknown voxel position after a scanner reboot (duration > 15min) (sub-30923).

### Supplementary Figure 3


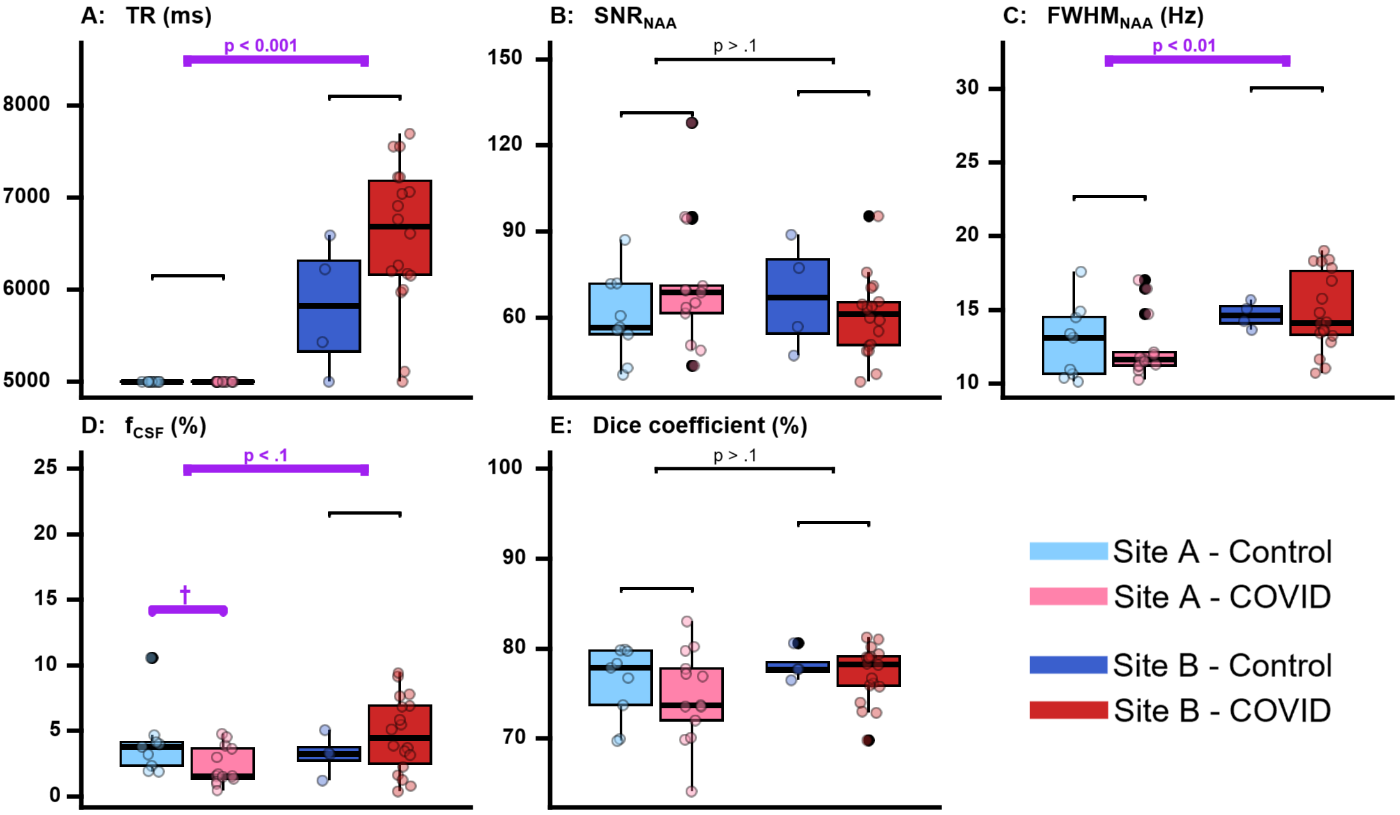


**Supplementary Figure 3:** Summary of acquisition parameters **(A),** spectral quality after removal of outliers and bad transients during Quality Assurance (**B-C**), and indicators of voxel placement (**D,E**). (**A**) TR differed significantly between sites. Differences in the revision of the International Electrotechnical Committee regulations in force at Site B meant that we could not achieve the 5s TR in all subjects there, whereas this was possible at Site A. We believe that this degraded the spectral quality achievable at Site B as seen in panels (**B,C**). Linewidths differed slightly between sites, which may be due to the availability of 3^rd^ order shims at Site A which were not available at Site B. (**D, E**) Neither the volumetric fraction of cerebro-spinal fluid (CSF) nor the Dice Coefficient showed significant differences between sites, confirming that we achieved consistent voxel placement and consistently good anatomical imaging for a robust segmentation. **Labels:** †, denotes a trend at 0.05 < p < 0.1, magenta p-values and brackets indicate p-values < 0.1 For detailed p-values see **Table 3** in main text.

### Supplementary Figure 4


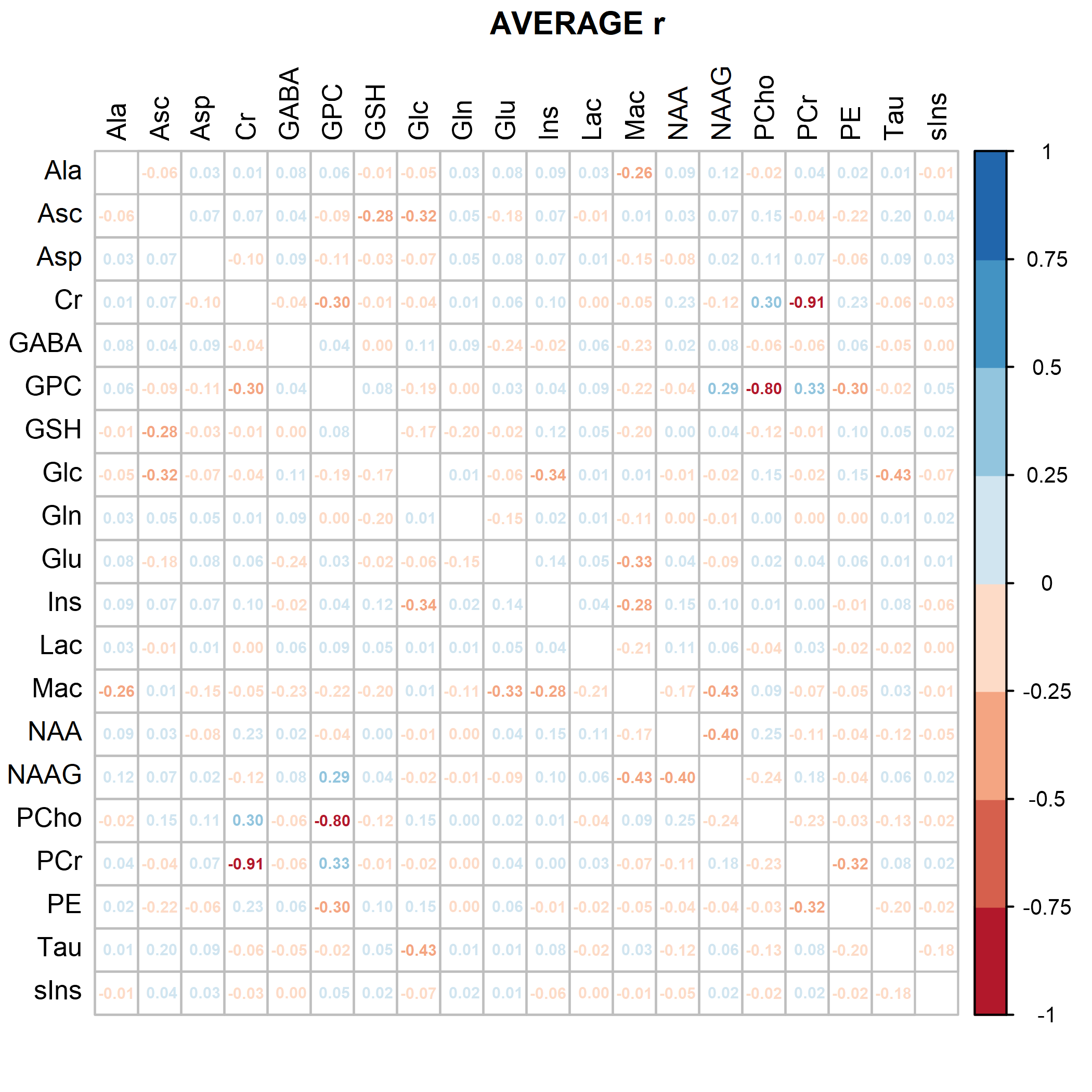


**Supplementary Figure 4: Average correlation matrix of the Metropolis-Hastings fitted samples across all participants.** Note that only GPC (glycerophosphocholine) and PCho (phosphocholine) as well as Cr (creatine) and PCr (phosphocreatine) had a Pearson correlation coefficient, r, of less than -0.7, and are therefore reported as combined concentrations, tCho and tCr, for further analysis. Meanwhile, we are reporting glutamate (Glu) and glutamine (Gln) separately, since these metabolites had a mean and lowest correlation coefficient of r_mean_(Glu, Gln)=-0.15 and r_min_(Glu, Gln)=-0.26, respectively for any spectra included in the final analysis.

### Supplementary Figure 5


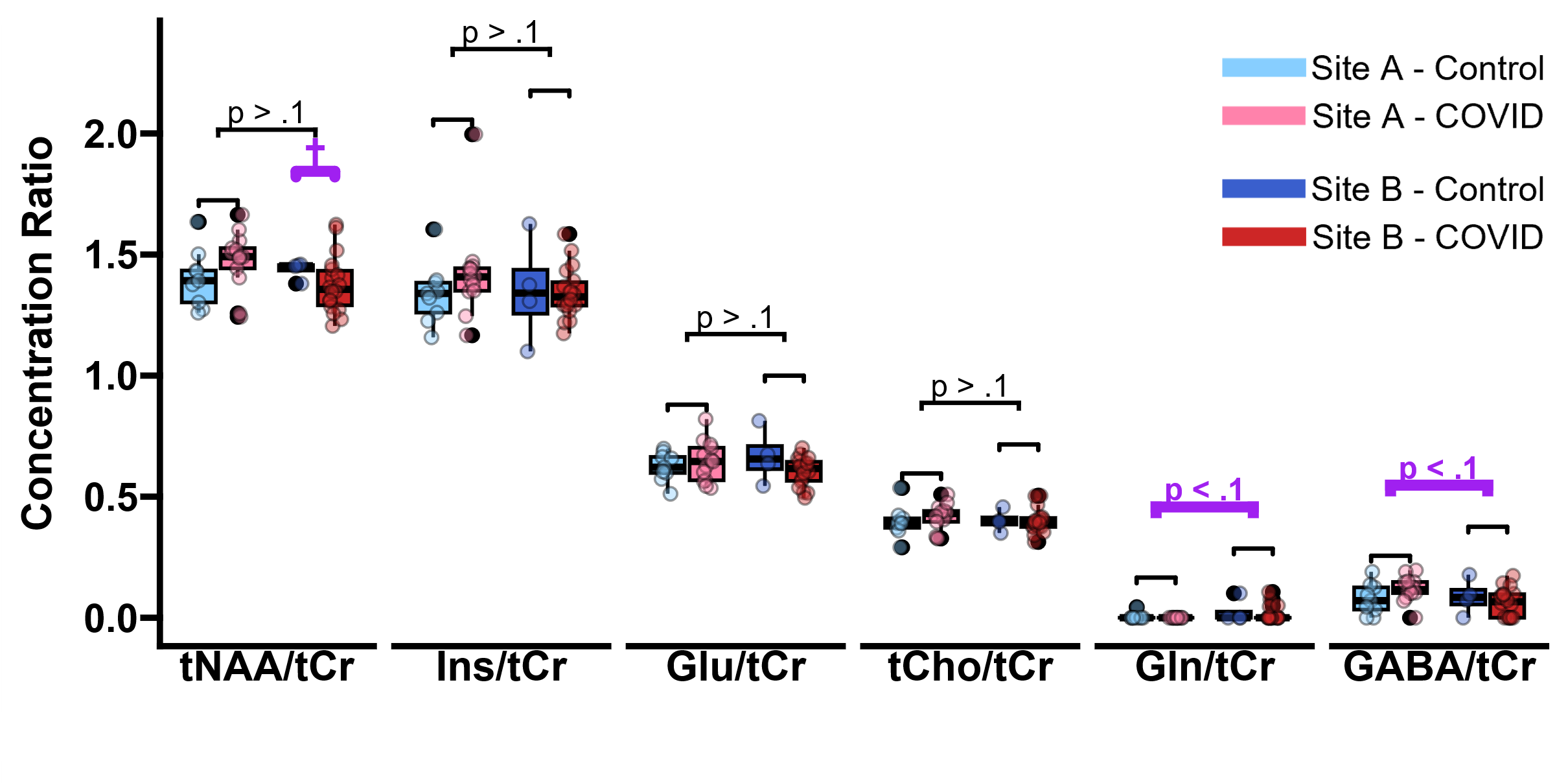


**Supplementary Figure 5:** Alternative analysis showing metabolite concentrations plotted as ratios relative to total Creatine (tCr). The systematic differences between sites are mostly removed (p (t-test) > 0.05) with only trends for Gln/tCr (p = 0.058) and GABA/tCr (p = 0.055). **Labels:** †, denotes a trend at 0.05 < p < 0.1. For detailed concentration ratios see **Supplementary Table 3**.

### Supplementary Figure 6


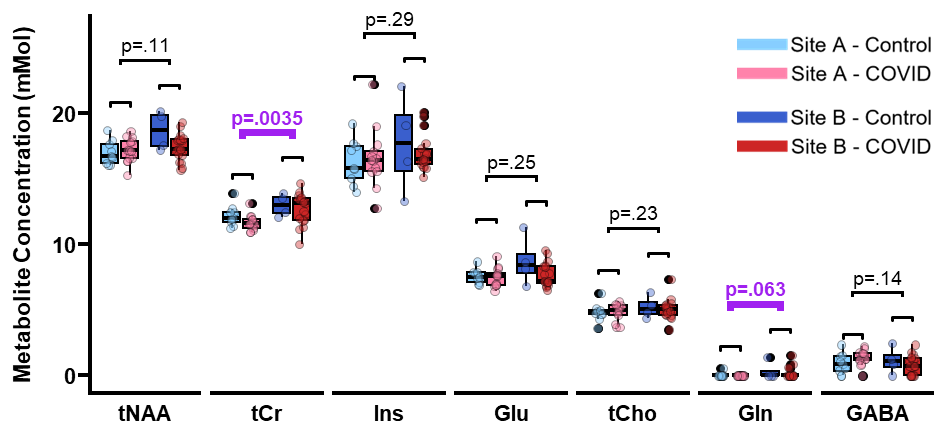


**Supplementary Figure 6:** Alternative analysis showing metabolite concentrations plotted corrected for T_1_ saturation effect losses, based on *cortical* metabolite T_1_ values. The systematic differences for tCr and Gln remain. **Labels:** magenta indicates p < 0.1, n.s., not significant.

### Supplementary Figure 7


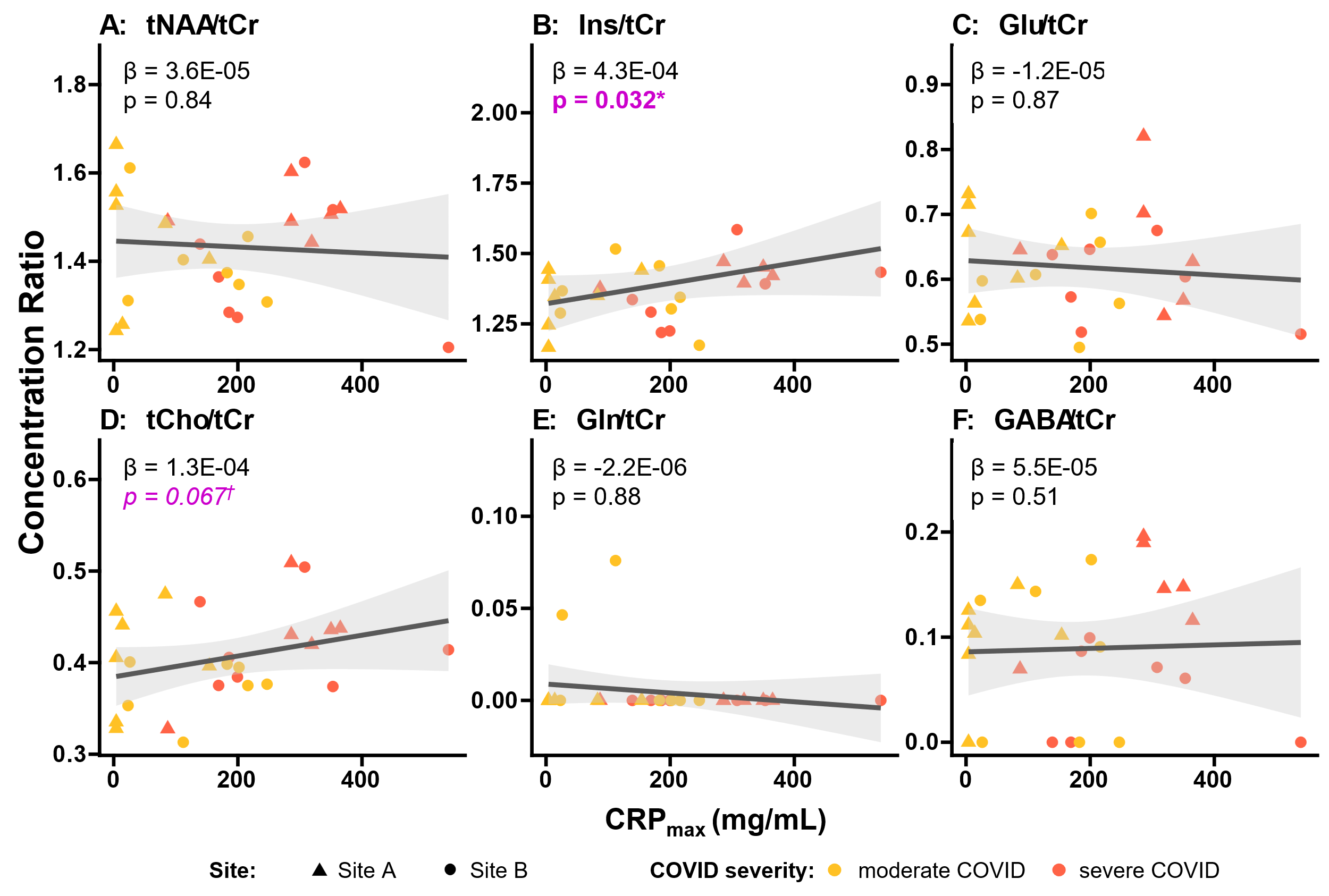


**Supplementary Figure 7:** Correlations of maximum CRP on tCr-referenced metabolite concentrations (i.e. [met]/[tCr]) pooled across both sites. Ins/tCr correlated significantly with CRP_max_ (β = 4.3E-04, 95% CI from 4.1E-05 to 8.3E-04, p = 0.032) and there was a trend for tCho/tCr (β = 1.3E-04, 95% CI from -9.8E-06 to 2.7E-04, p = 0.067) (model: [met]/[tCr] ~ CRP_max_ + (1|site) + (1|age) + (1|gender). This alternative analysis is therefore consistent with the main analysis using water-referenced concentrations as shown in Figure 4 of the main text.

### Supplementary Figure 8

**
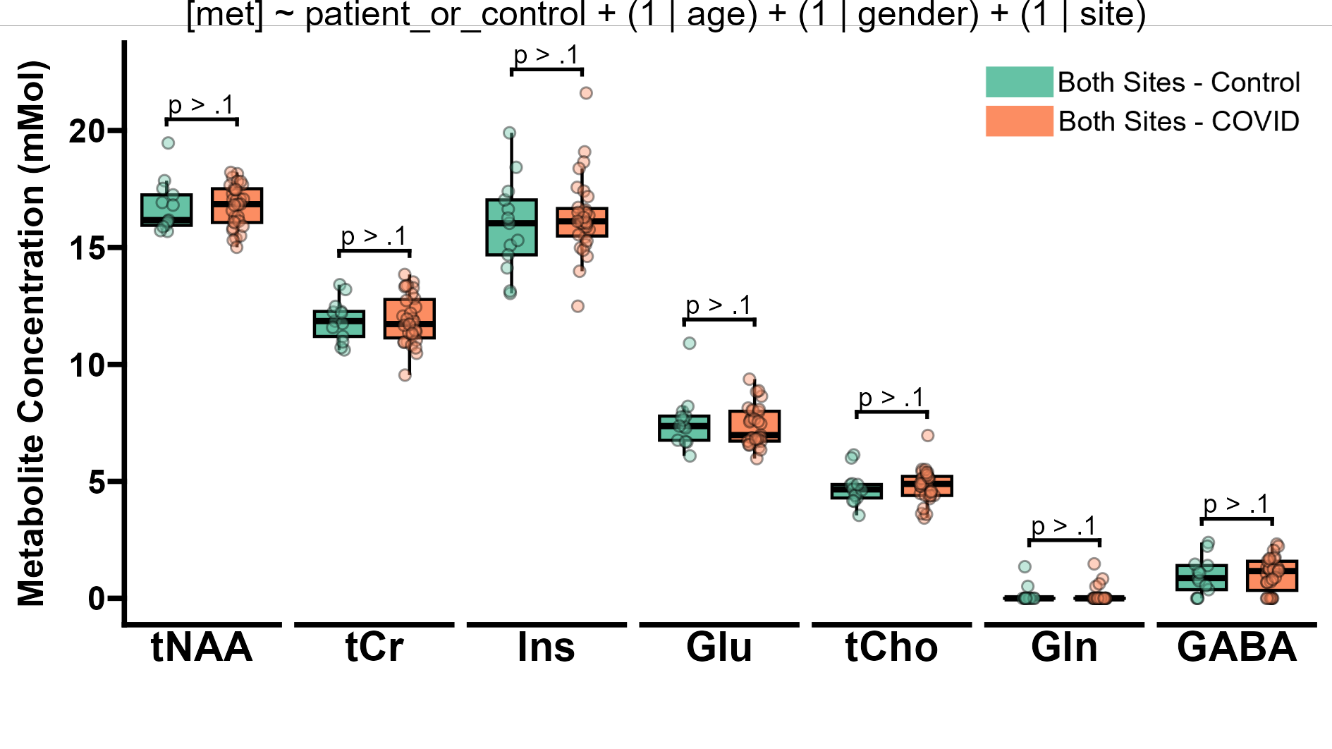
**

**Supplementary Figure 8: Metabolite concentrations at both sites analysed using the linear-mixed model defined in Eq (4) in the main text.** Metabolite concentrations are scaled relative to unsuppressed water, corrected for the voxel volume fraction of CSF, f_CSF_, and globally scaled by a standard water concentration, as described in eEq. (1) in row 3c of Supplementary Table 2. No significant differences were observed in controls vs patients after having corrected for site, age and sex differences.

### Supplementary Figure 9


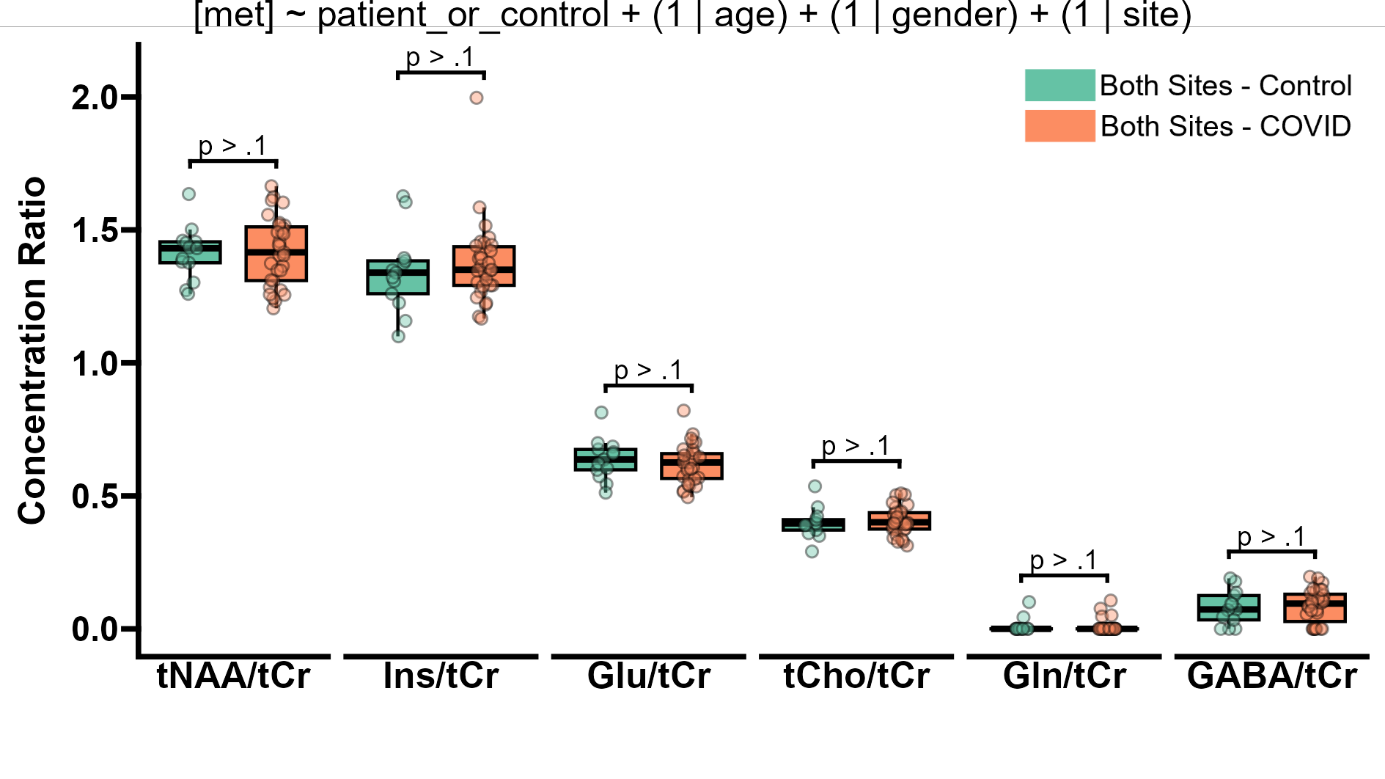


**Supplementary Figure 9:** In a joint analysis using a linear mixed model accounting for age, gender and site as random effects (model: [met]/[tCr] ~ patient + (1|site) + (1|age) + (1|sex)), no significant differences were detected between concentration ratios in patients and controls.

### Supplementary Figure 10


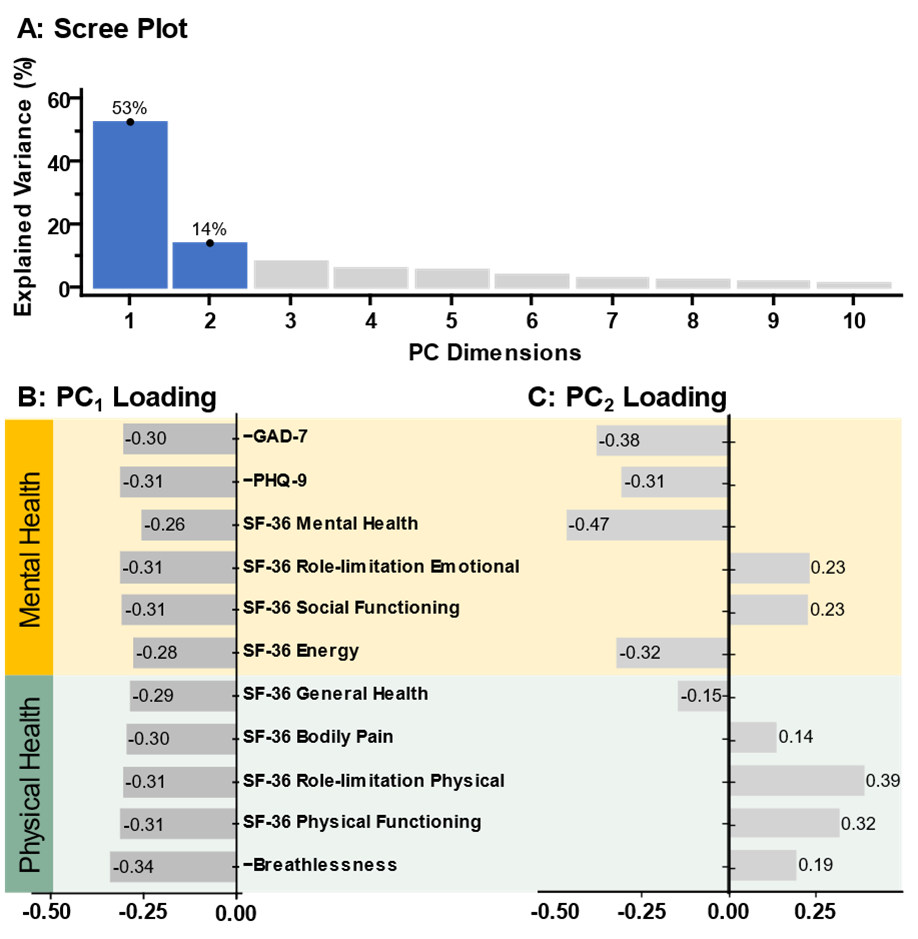


**Supplementary Figure 10: A**, Scree plot of principal component analysis. The first (PC_1_) and second (PC_2_) principal components explain 53% and 14% of the total variance, respectively. The third and higher components are each below a threshold of 10% and were not further investigated. **B, C** bar charts of the variable loadings of the first two components grouped by mental and physical health metrics. GAD-7, PHQ-9 and Breathlessness scores were inverted (multiplied by -1, indicated as −GAD-7, −PHQ-9, −Breathlessness) so that lower PC scores represented worse outcomes for all clinical metrics at follow-up. Note that PC_1_ is loaded evenly across all symptom metrics, while PC_2_ is loaded more strongly by mental health scores.

### Supplementary Figure 11


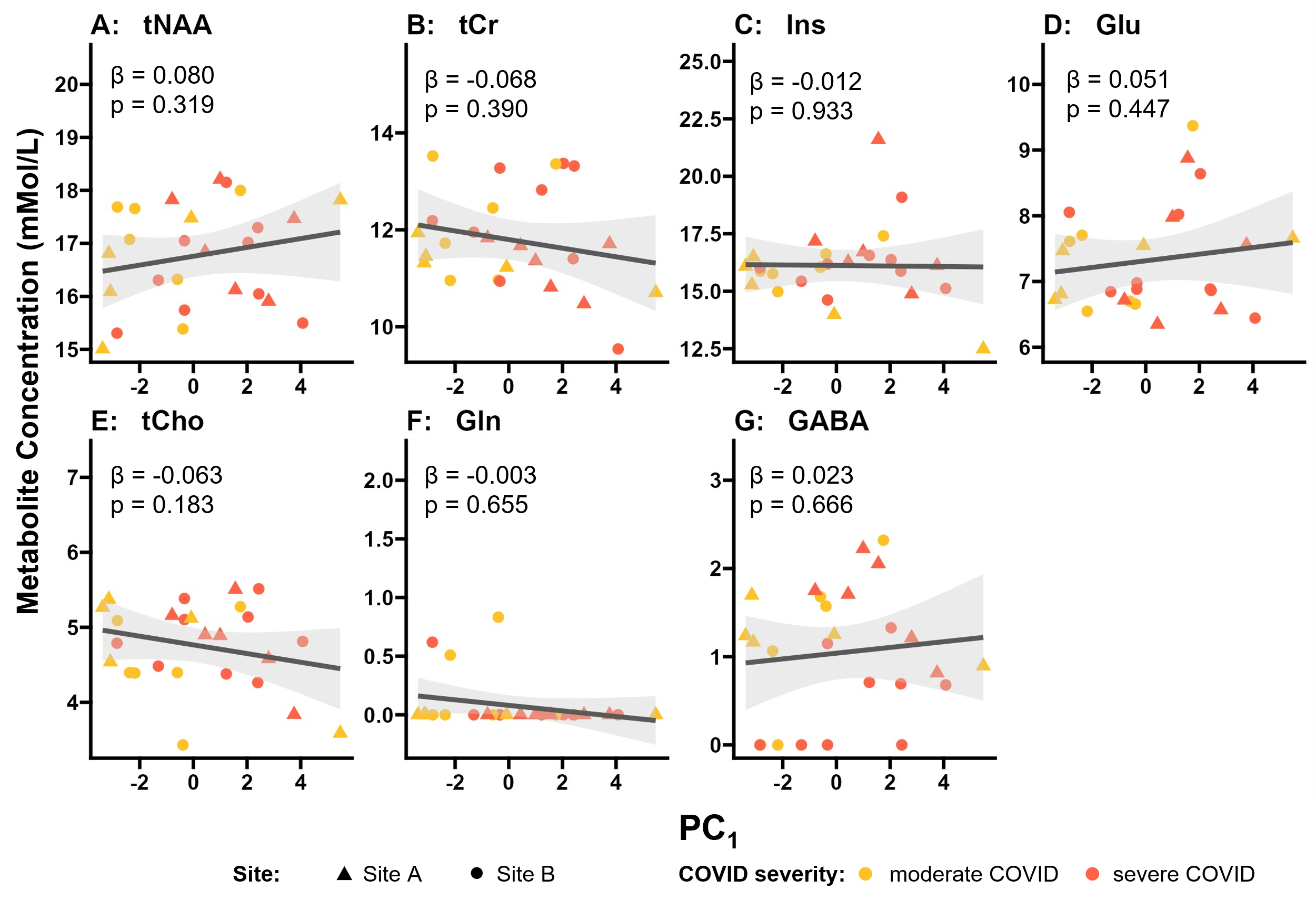


**Supplementary Figure 11:** Correlation of 1st principal component (PC­_1_) with metabolite concentration as determined by the linear mixed model from Eq (3) in the main text. There were no significant correlations between metabolite concentrations and PC_1_.

### Supplementary Figure 12

**

**

**Supplementary Figure 12:** Correlation of 2^nd^ principal component (PC_2_) with metabolite concentration as determined by the linear mixed model from Eq (3) in the main text. PC_2_ only correlated with Ins (β=0.564; 95% CI -1.043 to -0.086; *p*=0.023), suggesting that patients with overall poorer emotional wellbeing (lower PC_2_) have higher Ins.

### Supplementary Figure 13


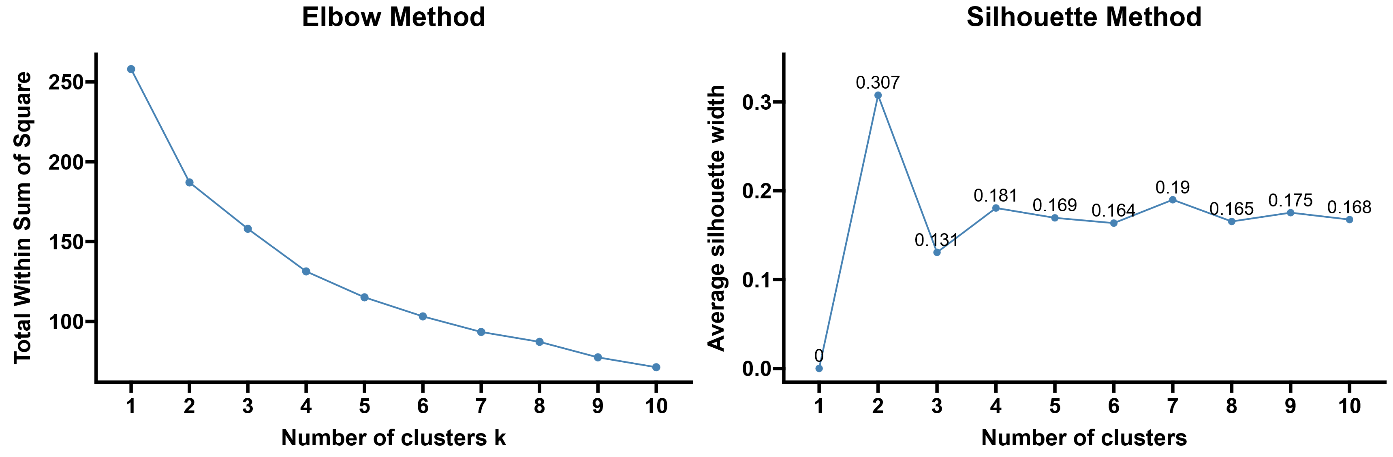


Supplementary Figure 13: k-cluster determination. (Left) the Elbow method does not show the typically distinctive sharp bend and as such the (right) Silhouette method was employed to determine the number of clusters. A “weak” clustering strength (mean silhouette width = 0.307) suggests the use of n=2 clusters for the k-means cluster analysis.

### Supplementary Figure 14


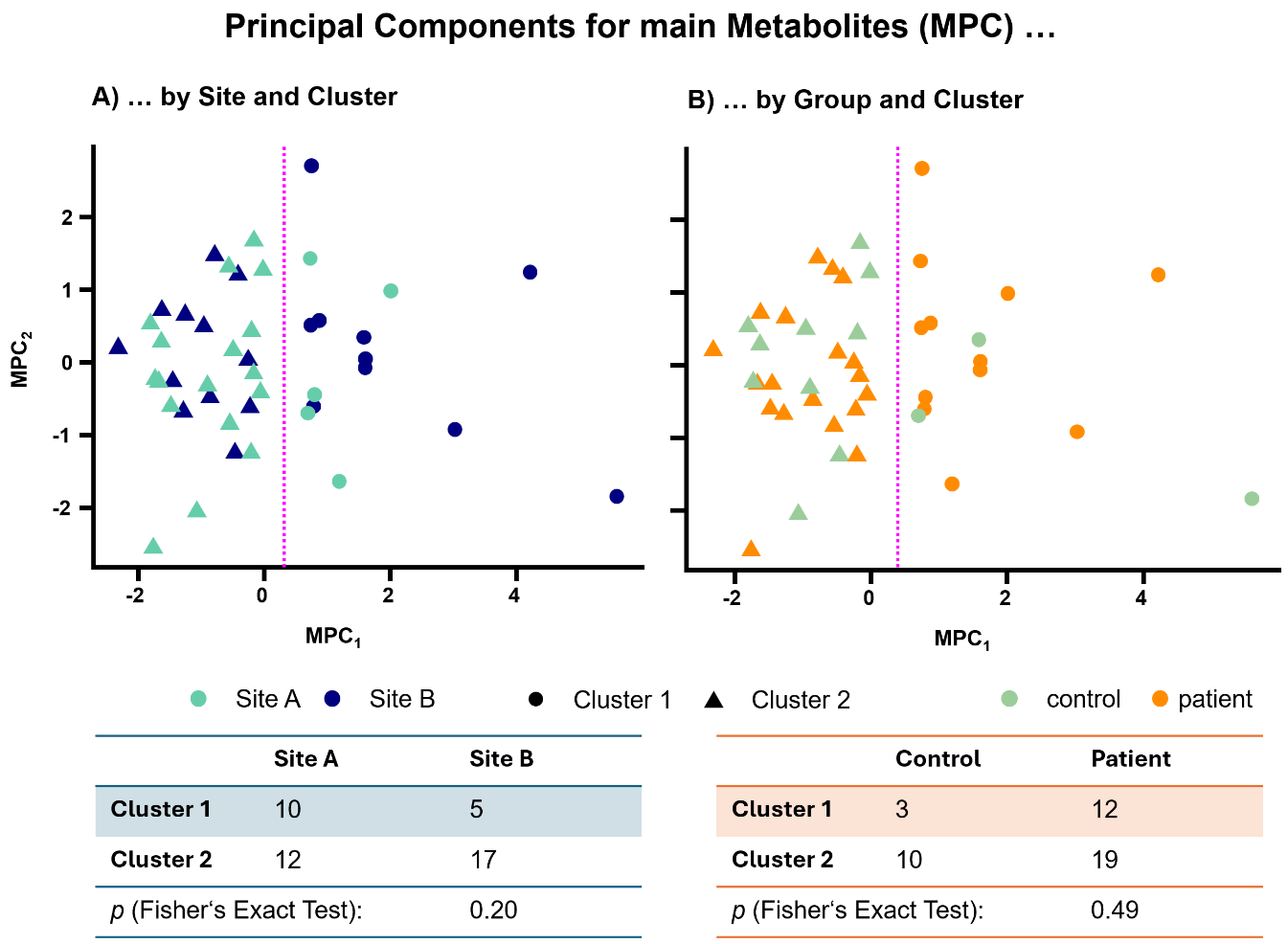


Supplementary Figure 14: Principal Components for main metabolites (MPC) following k-mean cluster analysis with n = 2.

### Supplementary Figure 15


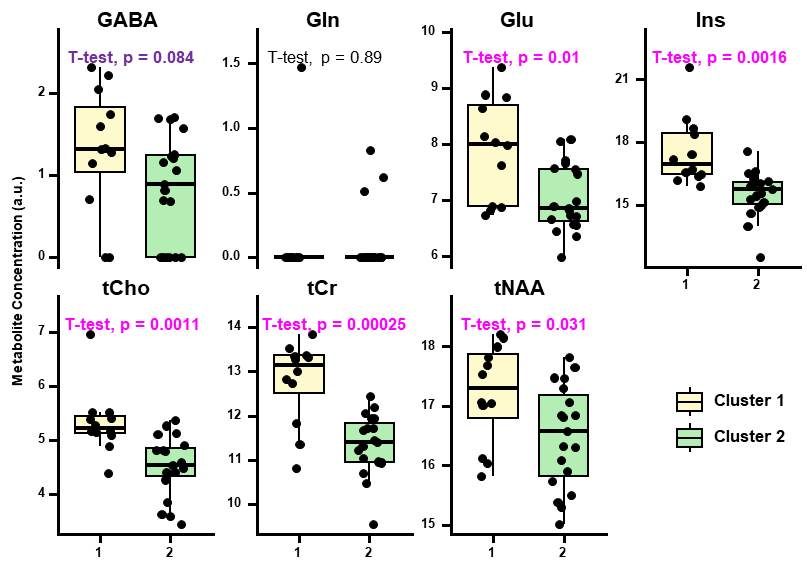


Supplementary Figure 15: Cluster analysis of main metabolite concentrations.

### Supplementary Figure 16


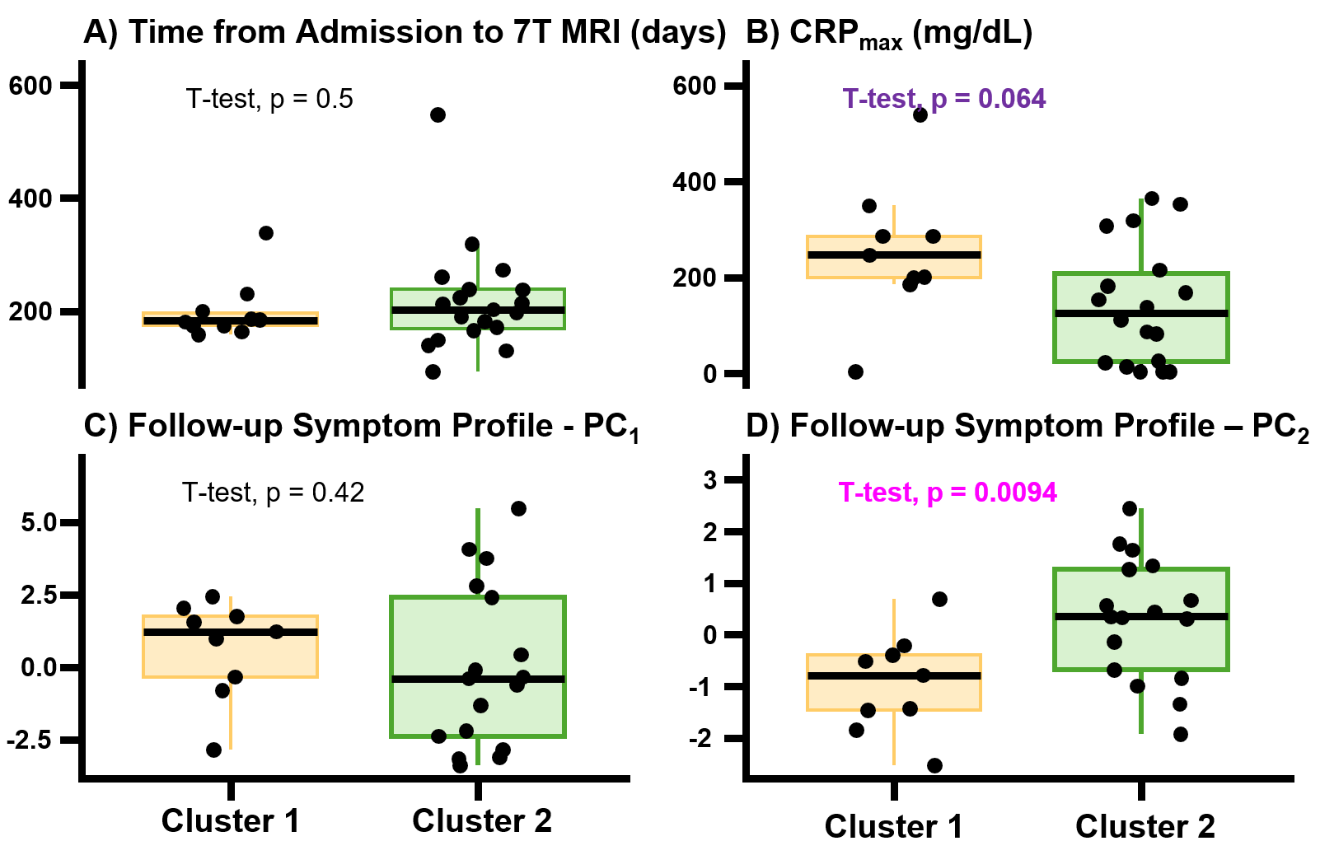


**Supplementary Figure 16**: Cluster comparison of key clinical metrics.

## Supplementary References

1. Chib S, Greenberg E (1995) Understanding the metropolis-Hastings algorithm. Am Stat 49:327

2. MacKay DJC (2003) Information theory, inference and learning algorithms. Cambridge University Press, Cambridge, England

3. Terpstra M, Cheong I, Lyu T, et al (2016) Test-retest reproducibility of neurochemical profiles with short-echo, single-voxel MR spectroscopy at 3T and 7T. Magn Reson Med 76:1083–1091

4. Papadakis M, Tsagris M, Dimitriadis M, et al (2023) Rfast: A Collection of Efficient and Extremely Fast R Functions

5. R Core Team (2023) R: A Language and Environment for Statistical Computing. R Foundation for Statistical Computing, Vienna, Austria

6. Kaufman L, Rousseeuw PJ (1990) Finding groups in data: An introduction to cluster analysis, 99th ed. John Wiley & Sons, Nashville, TN

7. Clarke WT, Mougin O, Driver ID, et al (2020) Multi-site harmonization of 7 tesla MRI neuroimaging protocols. Neuroimage 206:116335

8. Lin A, Andronesi O, Bogner W, et al (2021) Minimum Reporting Standards for in vivo Magnetic Resonance Spectroscopy (MRSinMRS): Experts’ consensus recommendations. NMR Biomed e4484

9. Gasparovic C, Song T, Devier D, et al (2006) Use of tissue water as a concentration reference for proton spectroscopic imaging. Magn Reson Med 55:1219–1226

10. UN World Health Organization (2020) WHO R&D Blueprint: Novel Coronavirus COVID-19 Therapeutic Trial Synopsis
